# Soft, Degradable, and Magnetic Microcarriers for Encapsulation and Guided Transport of Drugs and 3D Spheroids

**DOI:** 10.1002/adma.73735

**Published:** 2026-06-17

**Authors:** Xuan Peng, Lulu Song, Daryna Mruga, Veronika Bakhmat, Sergei Dzyadevych, Lin Guo, Shahrukh Shakeel, Rico Illing, Olha Bezsmertna, Xiaotao Wang, Nicholas R. Posselli, Sarthak Misra, Sandra Hauser, Jens Pietzsch, Klaus Kopka, Denys Makarov, Željko Janićijević, Xinne Zhao, Larysa Baraban

**Affiliations:** ^1^ Institute of Radiopharmaceutical Cancer Research Helmholtz‐Zentrum Dresden‐Rossendorf e.V. Dresden Germany; ^2^ Institute of Molecular Biology and Genetics of the NAS of Ukraine Kyiv Ukraine; ^3^ Institute of Ion Beam Physics and Materials Research Helmholtz‐Zentrum Dresden‐Rossendorf e.V. Dresden Germany; ^4^ Department of Biomechanical Engineering University of Twente Enschede The Netherlands; ^5^ Department of Biomedical Engineering University of Groningen and University Medical Centre Groningen Groningen The Netherlands; ^6^ Faculty of Chemistry and Food Chemistry School of Science Technische Universität Dresden Dresden Germany; ^7^ Else Kröner Fresenius Center for Digital Health Faculty of Medicine Carl Gustav Carus Technische Universität Dresden Dresden Germany

**Keywords:** cell spheroids, co‐encapsulation, controlled degradation, degradable hydrogel microcarriers, droplet microfluidics, magnetic navigation

## Abstract

Soft microcarriers hold great potential for biomedical applications, yet their translation is limited by the lack of controlled degradation, restricted capacity for co‐encapsulation of therapeutic and cellular cargos, long‐term biocompatibility, and scalable production challenges. Here, we introduce a new concept of discrete, degradable hydrogel microcarriers, produced via droplet‐based microfluidics and UV photopolymerization, designed to integrate these properties. These soft microcarriers enable co‐encapsulation of multiple species, including magnetic particles, drugs, and living cell spheroids, while allowing precise motion control over complex trajectories using external gradient magnetic fields. Their tunable degradation under physiological conditions ensures transient stability, controlled navigation, and safe clearance. This approach provides spatiotemporal control over cargo transport and release, enabling the microcarriers to function as systems with a controllable lifecycle. These multifunctional microcarriers represent a versatile platform for tissue engineering, minimally invasive therapies, and diagnostic monitoring, enhanced by the precise in situ navigation option.

## Graphical abstract

This work presents soft, degradable hydrogel microcarriers that combine magnetic responsiveness with the ability to host multiple therapeutic and cellular components. Produced by droplet microfluidics, the carriers maintain structural integrity during manipulation, permit controlled breakdown under physiological conditions, and enable guided motion for targeted biomedical tasks, offering new opportunities for delivery, repair, and monitoring.

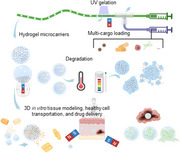

## 1 Introduction

Microcarriers have emerged as promising tools for next‐generation biomedical applications, including targeted delivery of medicines, tissue engineering, and diagnostic monitoring [1, 2, 3]. Recent advancements in functional materials have enabled the development of a novel class of soft carriers, actuated in response to external stimuli, such as light [4, 5, 6, 7, 8, 9, 10], ultrasound [11], solvents and chemical gradients [12, 13, 14], and electric [15] or magnetic [16, 17, 18, 19] fields. These carriers can achieve wireless operation and deterministic control while transporting therapeutic agents or cells in a minimally invasive manner, with the potential to improve precision and reduce systemic side effects [20]. Despite these advantages, their translation into clinical use has been limited. Current carriers often have limited capacity for co‐encapsulation, particularly of living components, lack controlled degradation, and raise concerns over long‐term biocompatibility [2]. These shortcomings prevent their reliable use in dynamic and physiologically complex environments.

**FIGURE 1:**
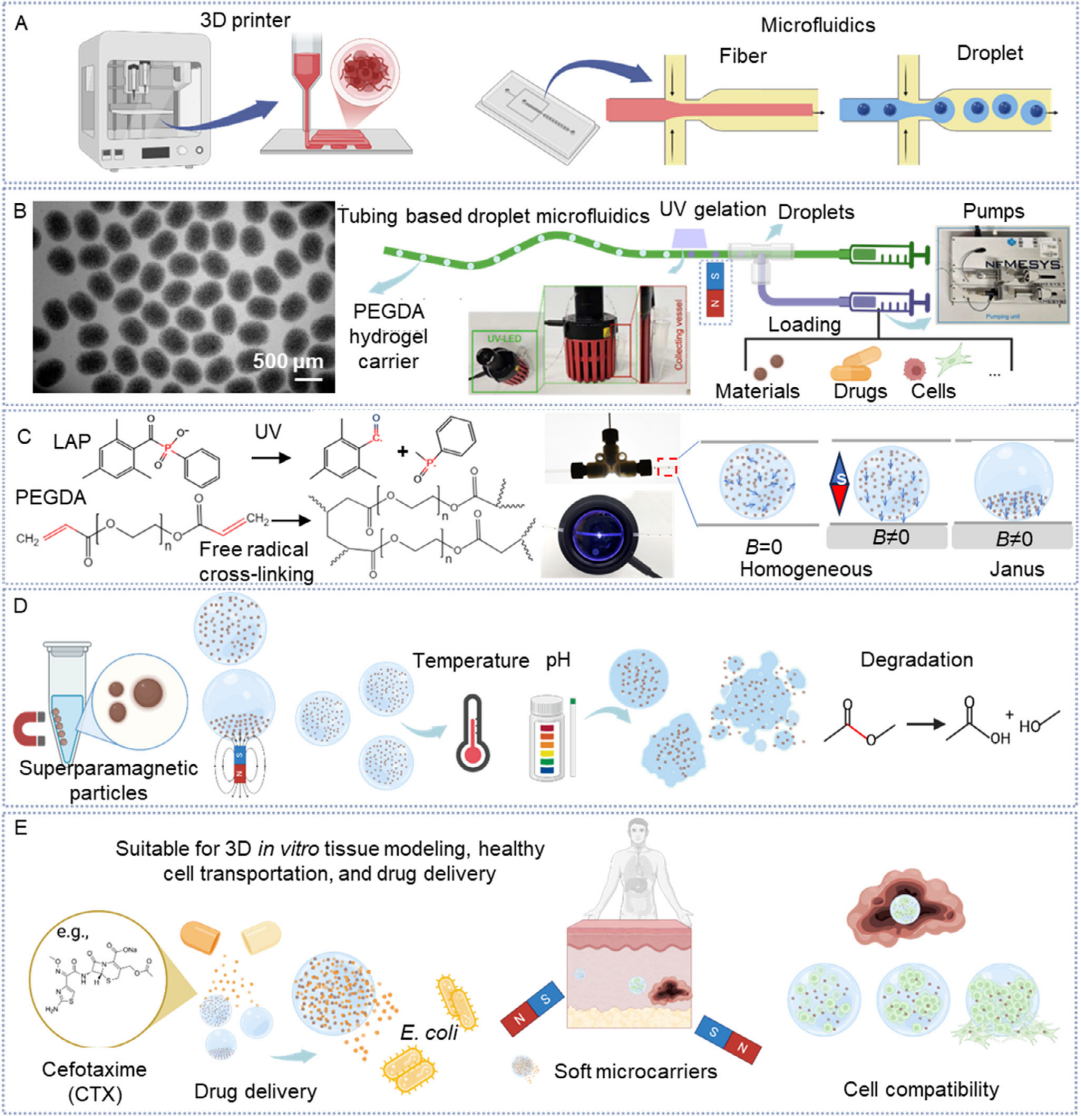
PEGDA microcarrier fabrication and applications. (A) Overview of soft functional materials and fabrication techniques, including 3D printing and microfluidics. (B) Schematic illustration of the generation of PEGDA microcarriers with superparamagnetic particles, including photographs of the microfluidic setup for the preparation of functional microcarriers and optical micrographs of PEGDA hydrogel microcarriers with magnetic particles. (C) Mechanism of PEGDA photopolymerization and illustrations of homogeneous and Janus‐type PEGDA hydrogel microcarriers. The latter are fabricated when hydrogel‐based magnetic microcarriers are photopolymerized in a gradient magnetic field. (D) Illustrations of the degradation of PEGDA hydrogel microcarriers influenced by temperature and pH. (E) Applications of PEGDA microcarriers in soft robotics, 3D cell culturing, healthy cell transportation, and drug delivery.

Magnetic soft carriers have demonstrated significant progress in design and fabrication, showing great potential for targeted therapeutic delivery and minimally invasive biomedical applications [21]. Recent advances in soft functional materials [22] and fabrication techniques—including 3D printing and microfluidics (Figure 1A)—have yielded impressive designs such as fibers, microcarriers, and Janus particles [17, 23, 24, 25, 26, 27, 28, 29]. For example, an acrylic elastomer‐based microcarrier integrated with ultra‐compliant pico‐force springs enabled non‐disruptive interactions with biological entities, though its operation was constrained by concomitant rotation of the entire body [30]. Recent studies have shown that microfluidics can be used to prepare multifunctional hydrogel‐based magnetic microfibers with disassembly functions under a rotating magnetic field [23], as well as droplet‐derived microrobots with self‐assembling functions for precise programmable cargo delivery [17]. These works demonstrated the versatility of microfluidics in microcarrier design, yet they lack studies on the simultaneous co‐encapsulation of different cargos, including, e.g., materials, drugs, and cells. Functionalities such as magnetic navigation, cargo transport, and carrier disassembly are often demonstrated separately rather than integrated within a single controllable system. Moreover, for safe, minimally invasive, and efficient medical use, microcarriers should be engineered to degrade, e.g., inside the body, after completing their programmed tasks [2]. However, precise and independent control over transport and release remains challenging in many existing systems.

In this work, we address this challenge by developing a multifunctional microcarrier platform that combines magnetic navigation, co‐encapsulation of therapeutic or biological cargos, and programmable hydrogel degradation within a single controllable system. To realize this concept, we fabricate soft, degradable, and magnetic hydrogel microcarriers through a high‐throughput droplet microfluidic system combined with UV photopolymerization of hydrogel materials (Figure 1B,C). By co‐encapsulating superparamagnetic particles (load up to 10% v/v) with drugs or living cells into poly(ethylene glycol) diacrylate (PEGDA) hydrogel microcarriers (diameter ∼500 µm), we create hydrogel microcarriers compatible with therapeutic delivery, living systems, and controlled degradation. We demonstrate precise control of carrier motion under external gradient magnetic fields (up to 2 T m^−1^) and their tunable degradation, with complete matrix dissolution achievable over timescales of several days to a week, depending on hydrogel composition, temperature, and pH (Figure 1D). The microcarriers enable efficient antibiotic delivery, with a stable antimicrobial effect on *Escherichia coli* (*E. coli*), directly correlated to the encapsulated drug concentration (Figure 1E). Hydrogel microcarriers also support mammalian cell culture and cancer spheroid growth, with successful demonstration of tissue delivery in vitro. By integrating magnetic guidance, programmable degradation, and application‐dependent cargo loading within a single system, the microcarriers establish a controllable lifecycle from guided transport to time‐regulated disassembly. Such spatiotemporal control over cargo delivery provides a versatile and sustainable strategy for minimally invasive therapies, regenerative medicine, and diagnostic monitoring.

## 2 Results

To provide a foundation for the multifunctional behavior presented in the following sections, we first detail the fabrication of degradable magnetic PEGDA microcarriers. This fabrication step is essential for achieving controlled magnetic actuation, tunable degradation, and co‐loading of therapeutic or cellular cargos.

### 2.1 Fabrication of Magnetic PEGDA Hydrogel Microcarriers

A large number (up to 10^3^) of sub‐millimeter hydrogel‐based magnetic soft microcarriers were fabricated in a liquid environment using a custom‐developed, T‐junction‐based microfluidic setup coupled with a UV gelation system (Figure 1B). The mechanical properties of the PEGDA hydrogel microcarriers can be tuned in a wide range of elastic moduli (10^2^–10^7^ Pa) [31]. Superparamagnetic dynaparticles (iron oxide particles) of 1 µm diameter based on polystyrene and iron oxide nanoparticles were co‐injected into the T‐junction to reach different final loading fractions of magnetic particles inside hydrogel microcarriers, enabling tunable magnetic responsiveness and controlled gelation for stable bead formation [32]. When filled with superparamagnetic microparticles, soft hydrogel microcarriers also reveal superparamagnetic behavior (Figure S1). After UV exposure (mean intensity: 18 or 34 mW cm^−2^, irradiation time: 0.2–8 s, Figure S2), due to the presence of hydrodynamic mixing of the components within emulsion droplets before polymerization, magnetic particles could be nearly homogeneously distributed within the volume of hydrogel microcarriers. In contrast, when integrating a permanent magnet into the setup near the UV polymerization area, Janus‐type microcarriers with an inhomogeneous distribution of magnetic particles across the bead could be fabricated as well (Figure 1C).

### 2.2 Impact of the Magnetic Particle Concentration and UV Exposure Time on the Fabrication of Microcarriers

**FIGURE 2:**
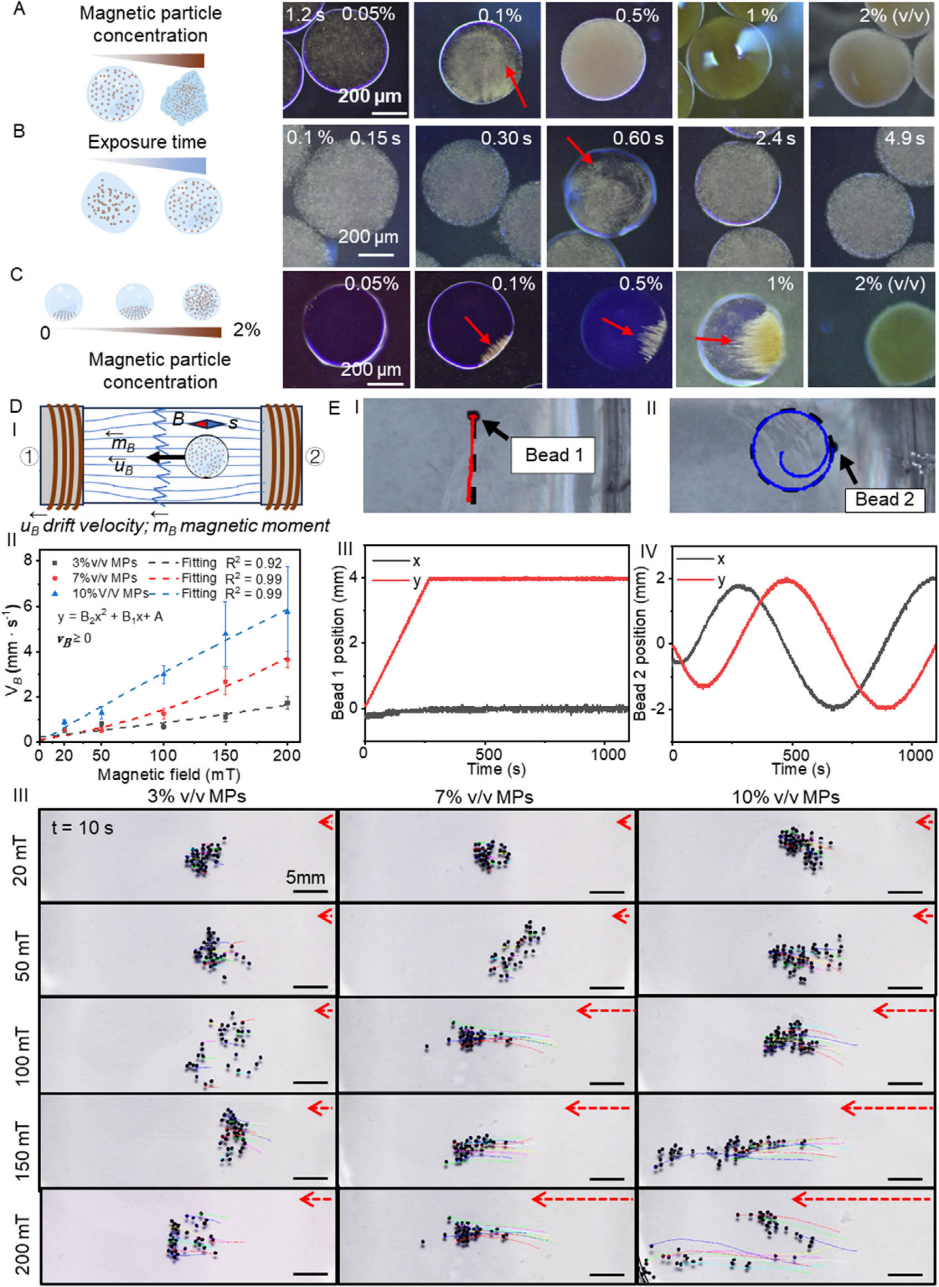
Impact of experimental parameters on the fabrication of magnetic microcarriers and motion of microcarriers in a gradient magnetic field. (A) Photographs of microcarriers generated with different concentrations of magnetic particles. (B) Photographs of microcarriers fabricated using different exposure times. (C) Representative images of Janus hydrogel microcarriers containing different concentrations of magnetic particles. Mean UV intensity: 34 mW cm^−2^, exposure time: 8 s. (D) Schematic of the velocity measurement experiment when magnetic microcarriers are exposed to a gradient magnetic field (I); Microcarriers tracking over time under different gradient magnetic fields (II), tracking paths of the same microcarriers at the 10‐s time stamp under different magnetic field strengths 20, 50, 100, 150, and 200 mT and corresponding gradients of 0.15, 0.39, 0.81, 1.3, and 1.7 T m^−1^, respectively (III); Dashed lines with an arrow illustrate the approximate displacements. Average velocities obtained from the tracking of different magnetic microcarriers containing 3, 7, and 10% (v/v) of magnetic particles under different magnetic field strengths. The diameter of the bead is about 500 µm. UV exposure time: 1.22 s, mean intensity = 34 mW cm^−2^. (E) Magnetic PEGDA microcarrier navigation within an electromagnetic system in a water bath. Programmed (dashed lines) and experimentally observed (solid lines) paths of the microcarriers (I&II); Time evolution of bead coordinates (III&IV).

After establishing a robust high‐throughput fabrication platform, we fabricated soft microcarriers containing different concentrations of magnetic particles (0%–10% (v/v)) under different UV exposure times (from 0.2 to 8 s, fixed mean intensity: 34 mW cm^−2^) (Figure 2). The concentration of encapsulated magnetic particles, photoinitiator content, UV light intensity, and UV exposure time play a crucial role in the gelation of soft microcarriers. In turn, the gelation efficiency crucially impacts the ability to immobilize magnetic particles within the hydrogel matrix and to achieve deterministic properties of soft hydrogel robots. The influence of UV exposure time and different concentrations of magnetic particles within droplets on the fabrication of hydrogel microcarriers is shown in Figure 2A–C. While a change in the microparticle concentration impacts the responsiveness of soft microcarriers to an external magnetic field, exposure time influences polymerization efficiency and mechanical properties of hydrogel microcarriers (Figure 2A). A very short exposure time and an excessively high concentration of injected magnetic microparticles could result in incomplete gelation (Figure 2A) due to absorption and scattering of light by magnetic microparticles within a hydrogel bead. To obtain soft microcarriers with uniform magnetic microparticle distribution, we varied the concentration of FeO_x_ magnetic particles and UV exposure time. The precursor mixtures of PEGDA and phenyl‐2,4,6‐trimethylbenzoylphosphinate (LAP) were prepared with different concentrations (0.05%–10% (v/v)) of magnetic microparticles with a diameter of 1 µm. The results show that concentrations of magnetic microparticles higher than 2% (v/v) (e.g., 3 and 4% (v/v)) start blocking the UV polymerization process (for the exposure time of 1.2 s) due to strong light absorption within the microcarriers (not shown). Droplets containing up to 10% (v/v) of magnetic particles begin to gel at around 0.2 s of UV exposure. To reliably obtain homogeneous microcarriers, the shortest polymerization time was about 2.4 s. When the exposure time is short (<2.4 s) and the gelation process is relatively slow, magnetic microparticles form undesirable mixing patterns inside hydrogel microcarriers (see red arrows in panels A and B, Figure 2). We also observed that, under all polymerization conditions, individual magnetic microparticles immobilized at the surface of hydrogel microcarriers were loosely attached to the PEGDA matrix and could leach into the solution. The leaching can be minimized by reducing the concentration of magnetic microparticles used for the fabrication of magnetic soft microcarriers to ≤1% (v/v).

Besides soft hydrogel microcarriers with a homogeneous distribution of magnetic particles, we also explored the process of generating Janus‐type hydrogel microcarriers (Figure 2C). For this purpose, we injected different concentrations of magnetic microparticles (2, 1, 0.5, 0.1, and 0.05% (v/v)) into microfluidic droplets and used a UV lamp (mean intensity: 34 mW cm^−2^) with an exposure time of 1.2 s. We placed a permanent magnet (NdFeB) within the UV polymerization area, resulting in an applied magnetic field of about 50 mT (at a distance of 2 mm from the fluidic tubing). The drift velocity of magnetic particles in a gradient magnetic field (Figures S3–S5) can be described by the equilibrium between the Stokes’ drag force and magnetic gradient force as discussed in a previous report [33]. The gradient magnetic field resulted in a quick attraction of superparamagnetic microparticles toward the region of high field gradient and thus their accumulation at one of the edges of hydrogel microcarriers (Figure 2C). We observed that magnetic particles aggregated on one side of the microcarriers, appearing to align with the magnetic field lines (red arrows shown in Figure 2C). For high loading fractions of magnetic microparticles (≥2% (v/v)), the structural inhomogeneity cannot be formed.

### 2.3 Microcarrier Navigation Along Complex Paths

Next, we analyzed the motion of magnetic soft microcarriers placed in an aqueous medium (Figure S9), under an applied external magnetic field gradient due to magnetophoresis [34] (Figure 2D). We then quantified their velocity by measuring the distance traveled over time intervals of ≥10 s. A schematic of the velocity measurement experiments is shown in Figure 2D (I). The microcarriers were loaded in a petri dish and placed between pole shoes of an electromagnet separated by about 10 cm. One coil of the electromagnet was not driven, while the magnetic field gradient was applied by driving the second coil. The magnetic field, reported in the following, was measured at the pole shoes of the second coil. In this respect, as the distance between the pole shoes is kept constant, the gradient magnetic field is proportional to the applied magnetic field. The biasing magnetic field is needed to induce a magnetic moment in the otherwise superparamagnetic microcarriers. Magnetic microcarriers with different loadings of magnetic microparticles reveal different responsivity to the same magnetic field. In particular, microcarriers with very low concentration of magnetic particles (e.g., 0.05%, data not shown) show no response to an external magnetic field. Magnetic microcarriers are subjected to a magnetic field of specific strength and gradient, inducing their movement to the region of a higher magnetic field gradient (in our experiment, from right to left; Figure 2D and Movie S1). The velocity of a magnetic bead with a homogeneous distribution of magnetic microparticles is found to scale quadratically with the strength of the applied magnetic field for low concentration of magnetic particles per carrier (Figure 2D (II)), which is in line with prior reports on the field‐driven displacement of superparamagnetic particles [35]. With the increase of the concentration of magnetic particles per carrier, we observe a transition from quadratic to linear scaling of the carrier velocity with the magnetic field strength (Figure 2D and Figure S10), which may arise from the combined influence of enhanced dipole–dipole interactions, local particle heterogeneity, and asymmetric particle distributions within the hydrogel matrix at elevated particle concentrations (Note S1) [17, 28].

Finally, we demonstrate the potential of microcarriers for individual navigation by autonomously moving two microcarriers along two distinct 2‐D paths simultaneously (Figure 2E and Movie S2). An electromagnetic system with nine coils [36] (Figure 2E (I)) was used to generate magnetic forces on the microcarriers to move them along the programmed paths while they floated on the surface of water (Figure 2E (I,II)) [37]. The path‐following algorithm successfully moved the microcarriers with desired velocities (Figure 2E (I,III)). Using this approach, one bead was guided along a straight line (Figure 2E (I,III)), while another one followed a circular trajectory (Figure 2E (II,IV)). Although each bead started off the target path, both converged toward their respective trajectories as they moved toward the endpoints. With this experiment, we demonstrate that our microcarriers can be navigated using automated systems for magnetic manipulation [18].

### 2.4 Programmable Degradation Behavior of Magnetic Soft Microcarriers

**FIGURE 3:**
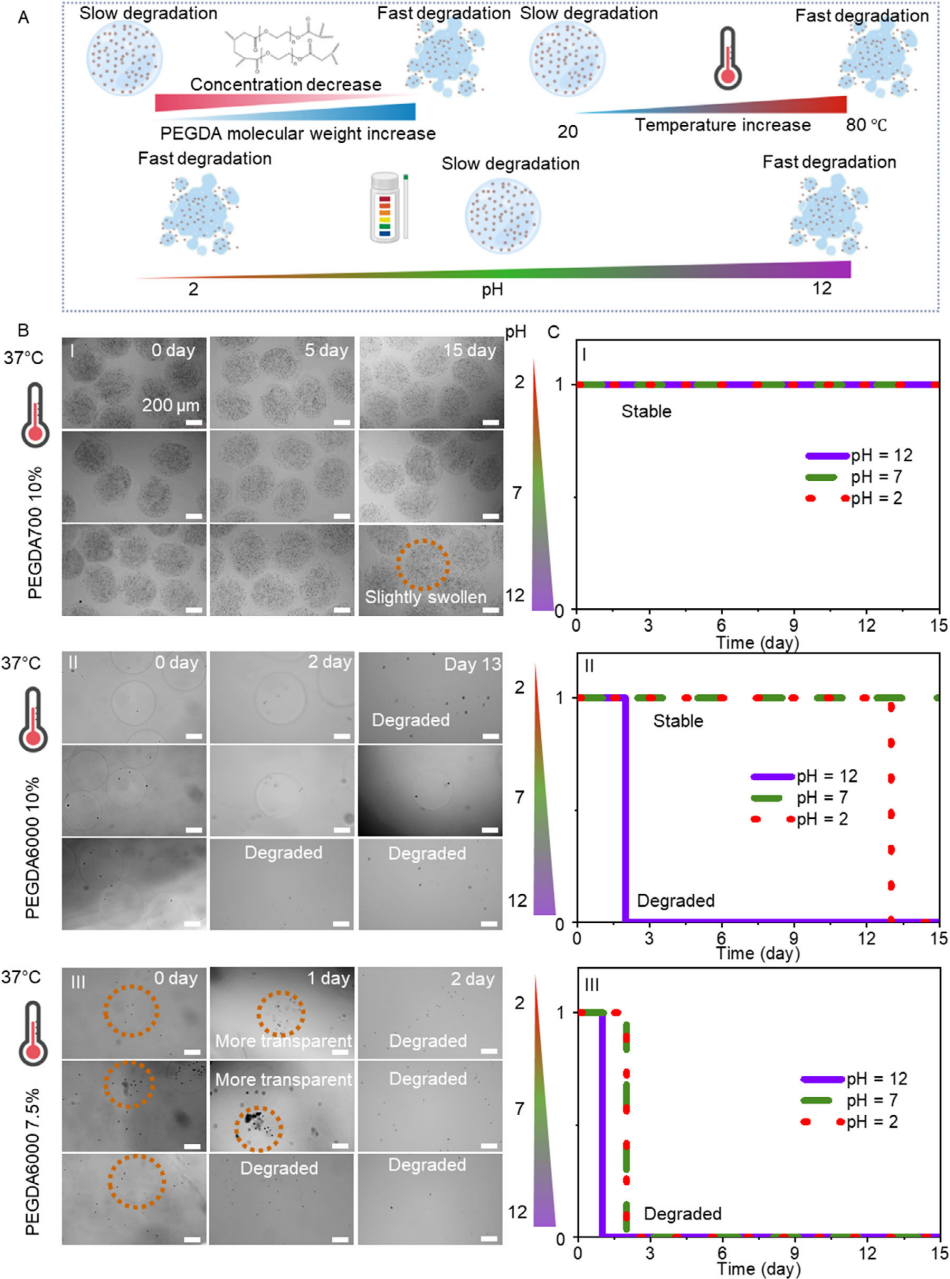
Controlled design of degradable magnetic microcarriers. (A) Illustration of the influence of monomer molecular weight, temperature, and pH on the degradation time of magnetic soft microcarriers. (B) Representative morphology of magnetic microcarriers cultured at 37°C under acidic (pH 2; top row), neutral (pH 7; middle row), and alkaline (pH 12; bottom row) conditions for different molecular weights of PEGDA monomer. Microcarriers PEGDA 700 10% (w/v) generated with 1% (v/v) of magnetic particles (diameter: 1 µm) (I) were fabricated under mean UV intensity of 18 mW cm^−2^ and irradiation time of 0.2 s; Microcarriers PEGDA 6000 10% (w/v) and 7.5% (w/v) generated with 2% (v/v) of magnetic particles (diameter: 10 µm) (II) were fabricated under mean UV intensity of 230 mW cm^−2^ and irradiation time of 2.5 s. Scale bar = 200 µm. (C) Degradation states of the microcarrier corresponding to the conditions shown in (B). In panel (C), “1” represents stability of the carriers with respect to degradation under the indicated conditions. “0” indicates that the carriers have degraded.

The diverse applications of magnetic microcarriers in biomedicine require tunable material properties from long‐term stability to rapid post‐delivery degradation. Here, we demonstrate the ability to tailor magnetic microcarriers on demand, tuning their longevity for longer (>15 days) or shorter (<3 days) lifetimes. To meet these requirements, microcarriers must be designed with appropriate materials and responsive degradation mechanisms. PEGDA hydrogels are well‐suited for this purpose, as they can degrade primarily through hydrolysis, enabling controlled lifetimes from rapid breakdown to extended stability [38]. The stability (slow degradation) of hydrogel microcarriers is governed by a number of factors, such as crosslinking density, molecular weight, and environmental conditions [39]. We investigated the degradation profiles of PEGDA‐based magnetic microcarriers (500 µm in diameter), and their stability and degradation behaviors are illustrated in Figure 3A. The stability and degradation of microcarriers were systematically investigated for PEGDA700 microcarriers fabricated with 1% (v/v) magnetic microparticles and PEGDA6000 microcarriers fabricated with 2% (v/v) magnetic microparticles, both exhibiting excellent gelling properties. Detailed experimental parameters are summarized in Table S1. These hydrogel microcarriers were dispersed in aqueous solutions with different pH values ranging from acidic (pH 2), through neutral (pH 7), and up to alkaline (pH 12). These samples were further divided and incubated at different temperatures starting from room temperature (RT), through 37°C, 60°C, and up to 85°C. The results of the degradation of magnetic microcarriers are summarized in Figure 3 and Figures S11–S13. The molecular weight and concentration of PEGDA monomer have a strong influence on the degradation profiles of the microcarriers. Overall, microcarriers generated with PEGDA700 exhibited greater stability than those fabricated with PEGDA6000 under all tested conditions. PEGDA700 magnetic microcarriers remained stable for several weeks at 37°C (Figure 3B,C (I)) across all three pH conditions, whereas PEGDA6000 microcarriers fabricated at 10% (w/v) were only relatively stable under neutral conditions (Figure 3B,C(II)). In contrast, PEGDA6000 microcarriers prepared at 7.5% (w/v) degraded within 2 days in all pH environments (Figure 3B,C(III)). Elevated temperatures make the strongest impact on the degradation profile of the microcarriers, leading to their complete dissolution already after days 2 to 5 (Figure 3 and Figures S11–S13). Furthermore, acidic and alkaline pH accelerate the degradation processes by causing additional swelling of the microcarriers (see circles in Figure 3B (I) and Figure S13), which increases the porosity of the material. It was observed that PEGDA700 microcarriers (UV intensity = 18 mW cm^−^^2^, irradiation time ≈ 0.2 s) degrade within approximately 10 days when incubated in a solution of pH 2 or pH 12 at 85°C (Figure S13). The degradation could originate primarily from the hydrolytic cleavage of ester bonds under both acidic and alkaline environments, which reduces crosslinking density and generates carboxyl (─COOH) and hydroxyl (─OH) groups, facilitating bulk erosion [38]. The process is subdivided into three phases, starting from the *swelling* (1) of the entire bead (increase of measurable diameter up to 10%–15%) and consequent increase of pore size in the hydrogel matrix, followed by the cleavage of ester bonds, i.e., *erosion* and *dissolution* [38] (2) of the matrix, and *release* (3) of magnetic microparticles into the solution, as illustrated in Figure S13.

Interestingly, the presence of magnetic microparticle inclusions additionally accelerates the degradation of magnetic microcarriers, compared to bare hydrogel microcarriers (Figure S14). This can be ascribed to a “premature” leaching of magnetic microparticles in the solution due to hydrogel swelling, producing µm‐sized voids in the hydrogel matrix that induce heterogeneous swelling and enhance infiltration of the aqueous solution. Superparamagnetic microparticles that are released in the solution, as shown in Figure S13 (Day 10), can potentially be collected using a permanent magnet and reused in the next hydrogel bead generation process.

### 2.5 Magnetic Carrier‐Mediated Drug Delivery for *E. coli* Eradication

**FIGURE 4:**
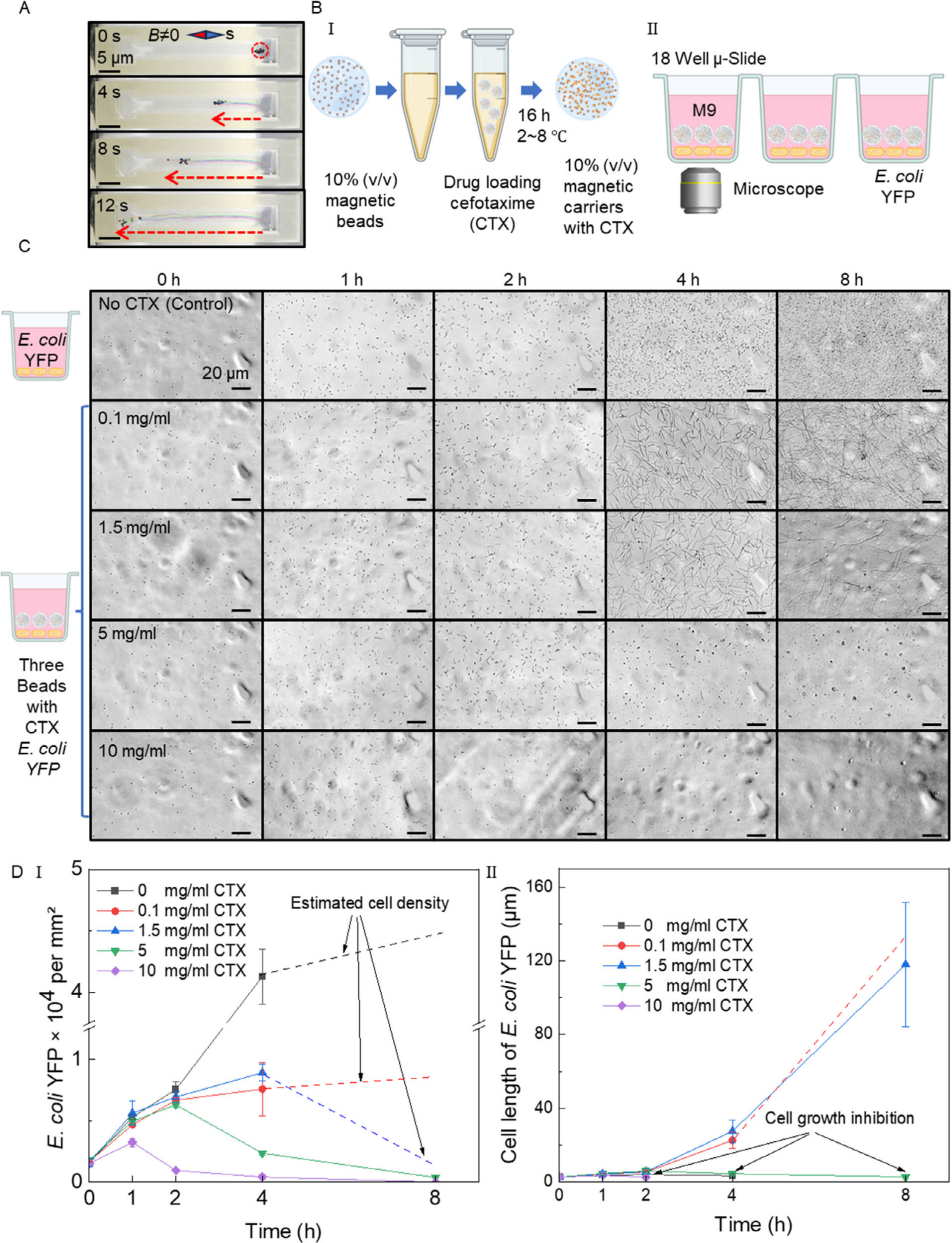
Magnetic carriers for antibiotic delivery and antibacterial effect. (A) Magnetic microcarriers (10% (v/v)) transport through a 5 mm channel under electromagnetic navigation (200 mT); Dashed lines with an arrow indicate the approximate displacement. (B) Schematic of 10% (v/v) magnetic carriers CTX loading (I) and releasing CTX for drug delivery and *E. coli* killing tests (II). (C) Proliferation and growth of *E. coli* YFP in the presence of magnetic carriers with different concentrations of CTX (0.1, 0.5, 1.5, 5, and 10 mg mL^−^^1^) at different time points. (D) Cell densities (I) and average length (II) of *E. coli* YFP (cells mm^−^^2^) quantified from panel C. Dashed lines indicate estimated trends where the numbers and lengths of *E.coli* cells were not precisely measurable, while the overall findings remain reliable.

To evaluate the antibiotic delivery potential, we first confirmed the transport of the β‐lactam antibiotic cefotaxime (CTX) loaded magnetic microcarriers (10% (v/v)) through a 5 mm wide 3D‐printed channel from chamber 1 (*t* = 0 s) to the intended “microbially contaminated” chamber 2 (*t* = 12 s) (Figure 4A). The magnetic microcarriers were successfully guided under a 200 mT magnetic field gradient, demonstrating the ability to navigate in confined environments. Then, the carriers were immersed in different concentrations of CTX for 16 h at 2°C–8°C to achieve antibiotic drug loading (Figure 4B (I)). The drug‐loaded magnetic microcarriers were then introduced into wells cultured with the gram‐negative and CTX‐sensitive *E. coli* YFP (*E. coli* strain – MG1655 galK::SYFP2‐FRT‐cat‐FRT with minimum inhibitory concentration (MIC) of 0.0625 µg mL^−1^) at an inoculum of OD_600_ = 0.01 A (5.1 × 10^6^ cells mL^−1^) (Figure 4B (II)). As the treatment progressed, the proliferation and morphology of the *E. coli* were significantly affected by increasing the concentration of CTX (Figure 4C and Figures S15–S17). In the absence of CTX (control group), the cell density rose to approximately 4 × 10^4^ cells mm^−^^2^ after 4 h and continued to increase thereafter (Figure 4D (I)), indicating normal bacterial growth. However, with the introduction of CTX, bacterial growth was significantly suppressed. When the CTX concentration was increased from 0.1 to 1.5 µg mL^−1^, the number of *E. coli* YFP cells reached only about 2 × 10^4^ cells mm^−^^2^ after 8 h of culture. Notably, under these conditions, cells exhibited a tendency to form filaments, a well‐known reaction to the antibiotic stress [40]. The microbial filaments reached lengths up to about 120 µm (Figure 4D (II)), which is in agreement with previous studies [37]. Further increasing the CTX concentration to 5 and 10 µg mL^−1^ caused a decline in cell growth and induced cell death, demonstrating a clear dose‐dependent antibacterial effect.

### 2.6 3D In Vitro Tissue Culturing

We selected the PEGDA monomer with a molecular weight of 6000 to form a soft extracellular matrix that is suitable for cell proliferation. As the use of larger PEGDA monomers causes the formation of a hydrogel with larger pores, magnetic particles of 10 µm in diameter, a size comparable to that of the cells, were incorporated to endow magnetic properties and prevent particle leakage from microcarriers.

**FIGURE 5:**
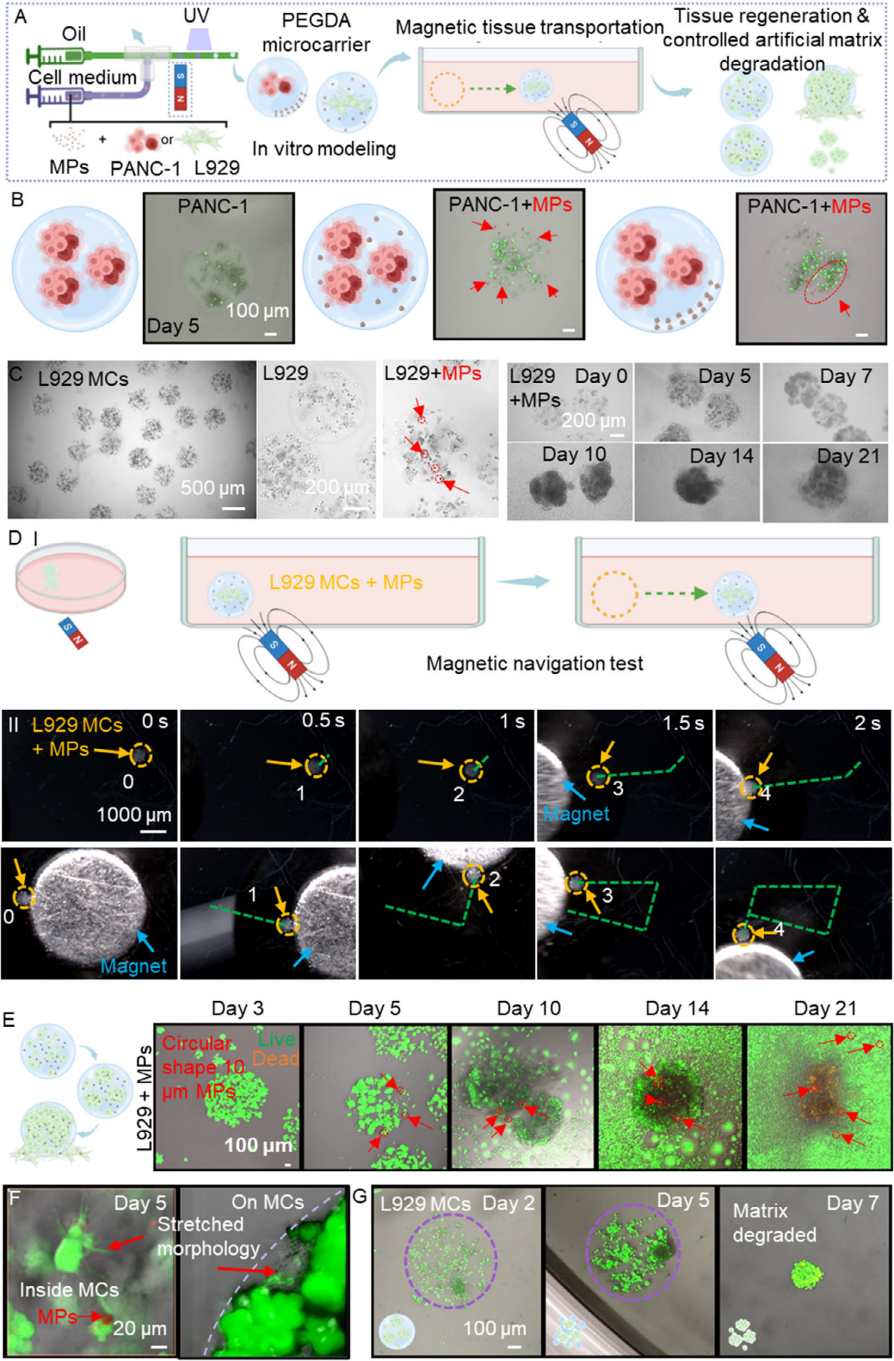
3D tissue culturing in PEGDA carriers. (A) Illustration of microcarriers with cell and magnetic particle co‐encapsulation, in vitro cell culturing, and magnetic delivery for tissue engineering studies. (B) Representative images showing PANC‐1 cells in PEGDA microcarriers generated with or without magnetic particles. (C) Representative images showing L929 cells in PEGDA microcarriers generated with or without magnetic particles (diameter: 10 µm) and cell proliferation in PEGDA Microcarriers with magnetic particles. (D) L929 microcarriers with magnetic particles moving under magnetic navigation. (E) Representative Live/Dead staining images. (F) Stretched morphology of L929 cells showing both inside and on the surface of the microcarriers. (G) L929 cultured in a degradable PEGDA matrix.

To demonstrate the versatility of our platform, we cultured the human pancreatic ductal adenocarcinoma cell line PANC‐1 and the mouse fibroblast cell line L929 within the PEGDA microcarriers (Figure 5A) and confirmed the successful generation of Janus microcarriers (Figure 5B). To assess biocompatibility and tissue‐transport capabilities of the microcarriers, and to highlight their potential use in tissue engineering and regenerative medicine, the mouse fibroblast L929 cell line, a standard cell line for biocompatibility and toxicity testing of materials and chemicals, was encapsulated into the PEGDA microcarriers (Figure 5C). No significant difference in L929 cell proliferation and viability was observed when cultured with or without magnetic particles (Figure 5E and Figures S18 and S19). The cells maintained high viability even after 2 weeks of culturing (Figure 5E). Clear cell death was observed after 20 days, likely due to nutrient and oxygen depletion in a confined 3D structure. L929 cells exhibited a typical filamentous, stretched morphology both inside and on the surface of the microcarriers (Figure 5F), indicating active extracellular matrix synthesis. After approximately 1 week of culturing, L929 cells migrated out of the microcarriers and continued proliferating on the plate. For implantation purposes in tissue engineering, it is generally desirable to retain cells at the implantation site while allowing dissolution of the artificial matrix. Hydrogel carriers generated using 7.5% (w/v) PEGDA showed matrix degradation within one week of culturing (Figure 5G). These results indicate that bead degradation can also be tailored when the carriers are loaded with cells, which may facilitate tissue implantation depending on the cell type. Furthermore, magnetic navigation tests using a permanent magnet demonstrated that the carriers containing cells could be guided along both linear and square trajectories (Figure 5D).

In addition, PANC‐1 cells were successfully maintained in the microcarriers, with no significant differences in proliferation or viability compared with controls (Figure S20). These results demonstrate that the platform supports not only fibroblast growth but also the formation and magnetic navigation of cancer spheroids. Given the rapid and controllable degradation of the hydrogel matrix, this system may be particularly valuable for developing implantable or migratory tumor models in animals. Overall, these findings highlight the biocompatibility, in vitro modeling capability, and tissue‐engineering potential of the degradable magnetic microcarriers.

## 3 Discussion and Conclusion

In this work, we present a versatile platform of degradable magnetic hydrogel microcarriers that integrate (i) co‐encapsulation of magnetic particles with biological or therapeutic payloads, (ii) controllable navigation along complex trajectories, and (iii) programmable or triggered degradation, which highlights the strength of droplet microfluidics for high‐throughput and highly tunable fabrication of microcarriers. Together, these capabilities enable a system‐level functionality in which transport, cargo loading, and degradation are integrated within a single platform.

A key advantage of this system is its ability to co‐encapsulate magnetic particles together with biological or therapeutic cargo, such as L929 fibroblasts, PANC‐1 cancer spheroids, or drugs. This direct co‐loading approach preserves cell viability within a protective hydrogel matrix and provides a magnetically responsive structure that can be guided remotely. Our demonstrations with fibroblasts and pancreatic cancer spheroids show that the microcarriers can support 3D tissue growth while being magnetically navigated with their living cargo. The ability of the microcarriers to transport viable tissue constructs further expands their potential applications, including human pluripotent stem cell culture, tissue regeneration, and other cell‐based therapeutic strategies [41].

Another central feature of the platform is that the microcarriers demonstrated robust navigation under externally applied magnetic field gradients, including the ability to follow predefined complex trajectories. This behavior supports the potential for automated closed‐loop navigation systems. The controllable guidance concept aligns with microrobotic navigation, where magnetic fields can be used to precisely position therapeutic or cellular cargo. In future translation efforts, the magnetic control demonstrated here could be integrated with clinically validated magnetic steering technologies [23, 42]. Such systems already provide precise magnetic field steering for intravascular devices and could be extended to microcarrier navigation, particularly for minimally invasive therapeutic interventions [43].

Moreover, the degradable hydrogel matrix ensures that, once positioned at the target location, the microcarrier gradually dissolves, releasing the embedded tissue and/or supporting the integration in vivo. This effectively enables the temporal regulation of cargo release following guided transport, linking degradation behavior to functional delivery outcomes. While this degradation originates from the intrinsic hydrolytic instability of PEGDA networks, in our system it is systematically engineered and tunable, rather than being a passive material property. Specifically, the degradation rate can be modulated by parameters such as polymer molecular weight, crosslinking density, and environmental conditions (e.g., pH and temperature), allowing the microcarriers to maintain structural integrity during transport and subsequently disassemble in a controlled manner. In addition, the incorporation of magnetic particles introduces structural heterogeneity and facilitates fluid penetration into the hydrogel network, further enabling programmable degradation over application‐relevant timescales. This feature is particularly relevant for regenerative medicine, subcutaneous implantation, and the development of in vivo models, as the degradation rate can be tuned to match the desired biological integration time. The microcarriers also exhibit accelerated degradation at elevated temperatures, enabling hyperthermia‐triggered dissolution at the target site [43]. This property can be further exploited for magnetic thermotherapy, where moderate hyperthermic conditions promote controlled hydrogel breakdown in a spatiotemporally defined manner, enhancing the precision and safety of tissue or therapeutic delivery.

Overall, this study demonstrates that degradable magnetic microcarriers fabricated via droplet microfluidics provide a powerful and flexible platform for biomedical applications. Importantly, beyond individual functionalities, the system establishes a programmable microcarrier lifecycle, in which magnetic navigation governs spatial positioning while engineered hydrogel degradation controls post‐delivery disassembly and cargo release, thereby enabling spatiotemporal control over cargo delivery. Besides these advantages, some limitations remain. The magnetic particles used in this study are not biodegradable, which may pose challenges for long‐term biocompatibility and clearance, and our demonstrations remain confined to controlled environments rather than clinically realistic conditions. Future work will require the incorporation of biodegradable magnetic materials, validation of navigation and degradation behavior in relevant biological models, and the conduct of in vivo safety and integration studies. These steps will be essential for translating the microcarriers from an experimental system to a deployable biomedical technology compatible with existing magnetic steering frameworks. Looking forward, the multifunctionality and precise fabrication of such soft hydrogel carriers open new opportunities for minimally invasive therapies, targeted drug delivery, and diagnostic monitoring. This approach could be extended to produce soft magnetic carriers of different geometries, such as rods and fibers, for a broader range of biomedical applications.

## 4 Experimental Section

### 4.1 Generation of Hydrogel Microcarriers Loaded with Magnetic Particles

Droplets were formed using a T‐junction (Figure 1B), where three tubes with an internal diameter of 500 µm met. By dosing a water‐based polymer solution and HFE oil (hydrofluoroether oil), small amounts of the two liquids were created as sub‐mm droplets depending on the dosing ratio. The dosing was realized using a NEMESYS pump (Cetoni, Nemesys) with two dosing modules. Each module was equipped with a 5 mL syringe containing one of the liquids. The water‐based polymer solution contained 10% (w/v) PEGDA (PEGDA 700, Sigma), 0.1% (w/v) LAP (Sigma), and various amounts of magnetic particles. The flow rate ratio had to be adjusted to realize a stable emulsion. Typical flow rates used in our experiments are 40 mL/h for HFE‐7500 (3M) and 2 mL/h for the aqueous solution. This flow rate ratio resulted in close‐to‐spherical droplets with a diameter of about 500 µm. After preparation, a series of droplets was stored in a storage unit, which was represented by a 1 m long tubing. For polymerization, the droplets were moved at a flow rate of 1.025 mL/h to a polymerization unit, where they were illuminated with UV light (365 nm wavelength; mean intensity: 18 or 34 mW cm^−2^) and exposed for up to 8 s. After exposure, the bead carriers were collected in an Eppendorf tube (5 mL). To fabricate Janus‐type magnetic microcarriers, a permanent magnet was placed below the tube before the droplet gelation process. The UV exposure time and a series of concentrations (2, 1, 0.5, 0.1, and 0.05% (v/v)) of superparamagnetic dynaparticles (diameter: 1 µm, Sigma) were tested to investigate different factors impacting the microsphere morphology. PEGDA6000 microcarriers were fabricated following a previously reported protocol, and 10 µm superparamagnetic dynaparticles Sigma) were incorporated [44]. The magnetic field decay of a magnet was measured using a hand Gaussmeter (HGM09s, MAGSYS magnet systeme GmbH.) from the magnet was fitted in Origin using a two‐parameter power function, *y*  =  *A* · (1 + *x*)^*B*^, which accounts for the finite field at the magnet surface and models the decay with distance through the adjustable exponent B (Figures S3–S5). In addition, the magnetic field intensity at the magnet surface was measured using a CMOS‐MagView device (software version 1.6.0.11b), which provided a surface field distribution over an approximately 4.5 mm region surrounding the magnet.

### 4.2 Measurement of the Velocity of Microcarriers in a Gradient Magnetic Field

A displacement of magnetic microcarriers in a gradient field of a permanent magnet was recorded with a camera (Nikon D7500). To quantify the velocity when exposed to a magnetic field of specific strength and gradient, magnetic microcarriers were loaded in a petri dish containing water (top) and HFE oil (bottom) phases (Figure S9). Microcarriers (stained with trypan blue 0.5% (w/v) overnight under 2°C–8°C) floated at the interface of the water and oil. The petri dish was placed under an adjustable magnetic field supplied by the DXWD‐80 electromagnet (Dexing Magnet Tech. Co., Limited; software: T 1.0.0.139). Different magnetic field strengths (40, 75, 150, and 240 mT) and corresponding gradients (0.3, 0.6, 1.3, 2 T/m) were applied to generate magnetic forces. Snapshot movies were recorded by a microscope for measuring the displacement of magnetic microcarriers in a gradient field.

### 4.3 Magnetic Navigation Demonstration

An electromagnetic system with nine coils [36]. was used to generate magnetic forces on the microcarriers to move them along the paths while they floated on the surface of water, similar to the work described in a previous report [37]. The path following algorithm moves the microcarriers at a desired velocity, ${\hat{v}}_{r}$, which is calculated as the weighted sum of a velocity component in the direction tangential to the closest point on the path to move the microcarriers forward, and a velocity component normal to the path to correct for error. The final velocity vector is calculated as

(1) $$v_{r}=v_{f}\frac{d\hat{n}+d_{r}\hat{t}}{\sqrt{d^{2}+d_{r}^{2}}}$$

where *v_f_* is the desired speed (we arbitrarily chose 15 µm/s), $\hat{n}$ is a unit vector normal to (pointing toward) the path, $\hat{t}$ is a unit vector tangential to the path, *d* is the distance from the bead to the nearest point on the path, and *d_r_* is a control parameter to weight between the normal and tangential components. The desired force to apply to the bead is

(2) $$f\;=k_{ff}\;\left(v_{f}+k_{p}\;\left(p_{d}\;-\;p\right)\right)$$

where *k_ff_* is a feedforward control gain used to compensate for drag forces, and *k_p_* is a proportional gain penalizing the difference in the bead's position, *P*, and the desired position, *P_d_*, which is calculated by numerical integration of ${\hat{v}}_{r}$. Finally, the calculation of the currents required to achieve the desired force is described in a previous report [36].

A camera suspended above the workspace of the electromagnetic system was used for image capture. OpenCV [45] was used to detect each of the microcarriers in the image using color thresholding and the Circle Hough Transform.

### 4.4 Degradation Study Under Different pH and Temperature

Preparation of acidic (pH 2), neutral (pH 7), and alkaline (pH 12) solutions: The acidic solution was prepared by diluting 30% hydrochloric acid (VWR BDH Chemicals) with deionized water. The neutral solution was prepared using phosphate‐buffered saline (PBS; Sigma–Aldrich) by dissolving one tablet in 200 mL of deionized water. The alkaline solution was prepared by solid sodium hydroxide (Sigma–Aldrich). Microrobotics fabricated under different UV exposure (mean intensity: 3.6–34 mW·cm^−^^2^; irradiation time: 0.2‐8 s) were maintained in acidic, neutral, and alkaline solutions (volume: about 3.4 mL each) by using a 24‐well cell culture‐treated plate (Corning Costar, REF 3526; flat bottom; polystyrene; non‐pyrogenic). To evaluate the temperature‐dependent degradation, the samples were incubated under three different temperature conditions: room temperature (approximately 22°C), 37°C using a shaker incubator (NB‐205Q), 60°C, and 85 °C using a laboratory oven (Heraeus T6).

### 4.5 Electromagnetic Navigation in a 3D Printed Channel

A microchannel was first designed using an open‐source 3D design platform (www.tinkercad.com/). The microchannel, with internal dimensions of 40 mm (length) × 5 mm (width) × 12 mm (height), was fabricated from clear resin using a 3D printer (Form 3B, Formlabs Inc., Somerville, MA, USA). A 200 µm‐thick glass coverslip (Menzel‐Gläser, 24 × 50 mm, No. 2 thickness, Thermo Fisher Scientific, Germany) was then bonded to the bottom of the printed microchannel with the same resin for printing and cured under natural sunlight for approximately 3 h. Subsequently, 10% (v/v) magnetic carriers pre‐stained with 0.5% (w/v) trypan blue were introduced into the channel for electromagnetic navigation tests using the same device described above.

### 4.6 *E. coli* Culturing and Drug Delivery Study

#### 4.6.1 Preparation of CTX‐Loaded Magnetic Carriers

Magnetic hydrogel carriers (10% v/v) were incubated in Cefotaxime (CTX; Sigma–Aldrich) solutions of 0.1, 0.5, 1.5, 5, and 10 mg mL^−^^1^ for approximately 16 h at 2°C–8°C.

#### 4.6.2 Surface Treatment of μ‐Slide

An ibidi μ‐Slide (18‐well, Gräfelfing, Germany) was pre‐coated with 150 µL of poly‐L‐lysine solution (Sigma–Aldrich) for 15 min to enhance bacterial adhesion. The solution was then discarded, and each well was rinsed twice with 200 µL of PBS.

#### 4.6.3 Bacterial Inoculation and Time‐Lapse Imaging

*Escherichia coli* YFP (*E. coli* YFP) cells were pre‐cultured overnight in M9 compound medium [40]. The culture was then diluted to an optical density at 600 nm of 0.01 (OD_600_ = 0.01 A, cell density = 5.1 × 10^6^ cells/mL). Fifty microliters of the diluted culture were added to each well. After placing the slide on an inverted microscope (Zeiss Axio Observer Z1) for 30 min to allow cell attachment, three CTX‐loaded magnetic carriers from each concentration were transferred into separate wells. One well without carrier served as a negative control. Bacterial growth was monitored by acquiring images every 20 min.

### 4.7 Cell Culturing

Mouse fibroblast L929 cells were purchased from Leibniz Institute DSMZ‐German Collection of Microorganisms and Cell Cultures. Frozen cells were thawed rapidly and resuspended in Dulbecco's modified Eagle's medium (DMEM, Gibco) supplied with 10% fetal bovine serum (FBS, Sigma–Aldrich), 1% penicillin/streptomycin (P/S, Biochrom) and 2 mm L‐glutamine (Sigma–Aldrich). Cells were maintained in T‐75 under 5% CO_2_ at 37°C. PANC‐1 human pancreatic cancer cells were purchased from ATCC (CRL‐1469) [46]. PANC‐1 cells were cultured in DMEM medium supplied with 10% FBS, 1% P/S. Cell culture medium was exchanged every 2–3 days. Upon reaching 80%–90% confluency, L929 cells were passaged using a standardized protocol. Briefly, cells were washed with 5 mL sterile PBS to remove residual serum and detached by incubating with 2 mL 0.05% trypsin‐EDTA (Gibco) at 37°C for 5 min. Trypsinization was halted by adding 8 mL complete medium. The cell suspension was centrifuged at 300 rcf for 3 min, and the pellet was then resuspended in fresh medium and seeded into new culture flasks.

### 4.8 Cell Culture in PEGDA Carriers and Live/Dead Assay

A solution of the complete L929 or PANC‐1 cell culture medium containing 10% (w/v) PEGDA (Mn 6000 Sigma–Aldrich), 0.1% (w/v) LAP, and L929 cells (c.a. 1.8×10^7^ cells·mL^−1^) was used as the water phase (WP). Mineral oil (Sigma–Aldrich) was used as the oil phase (OP). The flow rates for WP and OP were q_WP_ = 5.5 µL·min^−1^ and q_OP_ = 110 µL· min^−1^. The applied UV light intensity was 0.23 W·cm^−2^ and the exposure time was ∼2.55 s. The culturing cell medium was refreshed every 2–3 days. Cell proliferation was monitored using an optical microscope (Axio Observer, Zeiss). To obtain the degradable hydrogel matrix, cell culture medium containing 7.5% (w/v) PEGDA and 0.075% (w/v) LAP. To generate the microcarriers with both cells and magnetic particles, 2 or 3% (v/v) 10 µm magnetic particles (microparticles based on polystyrene, #49664, Sigma) were added to the previously mentioned WP. 1 mL magnetic particles were centrifuged at 300 rcf for 3 min, followed by washing with 99% ethanol and PBS for 3 times, respectively. The magnetic particles were resuspended in 1 mL 1× PBS and stored in a 4°C fridge before use. A conical permanent magnet was used for the magnetic navigation test. The magnetic field decay and the field distribution over an approximately 4.5 mm region surrounding the navigation magnet were measured using the same methods described above (Figures S6–S8). The movement of the microcarriers was recorded using a Keyence digital microscope (VHX‐7000).

Calcein AM/ propidium iodide (PI) live/dead staining was used to track cell viability in PEGDA hydrogel microcarriers. The microcarriers were first stained with calcein AM for 1 h (the first 1 week of culturing) or 3 h (after one week of culturing), and then with PI for 1 min. The solutions of staining chemicals were prepared following the instructions from the supplier (VWR (Corning)). Images were recorded using a confocal fluorescence microscope (Olympus IX83). The emission wavelengths of the lasers are 488 and 561 nm, with corresponding band‐pass filters of 500–540 nm and 570–620 nm, respectively. Cell viability was quantified using ImageJ (version v1.54i). A custom macro was used to quantify the average intensity by manually adjusting the threshold.

### 4.9 Statistical Analysis

Data are reported as mean ± SD. *p*‐values were calculated using the Two‐way ANOVA combined with the Bonferroni multiple comparison test using GraphPad Prism 10.6.1 depending on the analyzed dataset. Differences between experimental groups were considered as significant when ns(0.1234), ^*^ *p* < 0.0332, ^**^ *p* < 0.0021, ^***^ *p* < 0.0002, and ^****^ *p* < 0.0001, *n* ≥ 3.

## Author Contributions

Conceptualization: XP, ZJ, DM, LB, methodology: XP, SD, SS, RI, OB, NRP, SM, SH, ZJ, XZ, investigation: XP, LS, DM, VB, SD, LG, OB, XW, NRP, SM, SH, visualization: XP, LS, DM, NRP, XZ, funding acquisition: DM, LB, JP, KK, project administration: DM, LB, JP, KK, supervision: DM, LB, writing – original draft: XP, LS, LB, SS, DM, writing – review and editing: ZJ, XZ, DM, LB, JP, KK.

## Funding

This work was supported by the German Research Foundation (DFG; project SCHU 3512/2‐1, BA 4986/10), European Union in the frame of the project BIONANOSENS (grant agreement #951887), project REGO (grant agreement #101070066), ERC project 3DmultiFerro (grant agreement #101141331), ERC project ImmunoChip (grant agreement # 101045415).

## Conflicts of Interest

The authors declare no conflicts of interest.

## Supporting information




**Supporting File 1**: adma73735‐sup‐0001‐SuppMat.docx.

**Supporting File 2**: adma73735‐sup‐0002‐MovieS1.avi.

**Supporting File 3**: adma73735‐sup‐0003‐MovieS2.mp4.

## Data Availability

The data that support the findings of this study are available from the corresponding author upon reasonable request.

## References

[1] M. Ye , Y. Zhou , H. Zhao , et al., “A Review of Soft Microrobots: Material, Fabrication, and Actuation,” Advanced Intelligent Systems 5 (2023): 2300311, 10.1002/aisy.202300311.

[2] S. Chen , D. E. Fan , P. Fischer , et al., “A Roadmap for Next‐Generation Nanomotors,” Nature Nanotechnology 20 (2025): 990–1000, 10.1038/s41565-025-01962-9. 40750672

[3] P. Wrede , E. Remlova , Y. Chen , X. L. Deán‐Ben , M. Sitti , and D. Razansky , “Synergistic Integration of Materials in Medical Microrobots for Advanced Imaging and Actuation,” Nature Reviews Materials 10 (2025): 888–906, 10.1038/s41578-025-00811-4.

[4] G. Vizsnyiczai , G. Frangipane , C. Maggi , F. Saglimbeni , S. Bianchi , and R. Di Leonardo , “Light Controlled 3D Micromotors Powered by Bacteria,” Nature Communications 8 (2017): 15974, 10.1038/ncomms15974. PMC5493761 28656975

[5] T. Huang , V. R. Misko , S. Gobeil , et al., “Inverse Solidification Induced by Active Janus Particles,” Advanced Functional Materials 30 (2020): 2003851, 10.1002/adfm.202003851.

[6] T. Huang , V. Misko , A. Caspari , et al., “Electrokinetic Janus Micromotors Moving on Topographically Flat Chemical Patterns,” Communications Materials 3 (2022): 60, 10.1038/s43246-022-00282-y.

[7] T. Huang , B. Ibarlucea , A. Caspari , et al., “Impact of Surface Charge on the Motion of Light‐Activated Janus Micromotors,” The European Physical Journal E 44 (2021): 39, 10.1140/epje/s10189-021-00008-x. PMC7987638 33755813

[8] T. Huang , S. Gobeil , X. Wang , et al., “Anisotropic Exclusion Effect between Photocatalytic Ag/AgCl Janus Particles and Passive Beads in a Dense Colloidal Matrix,” Langmuir 36 (2020): 7091–7099, 10.1021/acs.langmuir.0c00012. 32011149

[9] X. Wang , L. Baraban , V. R. Misko , et al., “Visible Light Actuated Efficient Exclusion between Plasmonic Ag/AgCl Micromotors and Passive Beads,” Small 14 (2018): 1802537, 10.1002/smll.201802537. 30238700

[10] D. W. Kim , P. Wrede , A. Rodríguez‐Camargo , et al., “Upconversion Nanoparticle‐Covalent Organic Framework Core‐Shell Particles as Therapeutic Microrobots Trackable With Optoacoustic Imaging,” Advanced Materials 37 (2025): 2418425, 10.1002/adma.202418425. 40052638 PMC12691905

[11] V. R. Misko , L. Baraban , D. Makarov , et al., “Selecting Active Matter According to Motility in an Acoustofluidic Setup: Self‐propelled Particles and Sperm Cells,” Soft Matter 19 (2023): 8635–8648, 10.1039/d3sm01214j. 37917007

[12] M. Wehner , R. L. Truby , D. J. Fitzgerald , et al., “An Integrated Design and Fabrication Strategy for Entirely Soft, Autonomous Robots,” Nature 536 (2016): 451–455, 10.1038/nature19100. 27558065

[13] L. Baraban , D. Makarov , R. Streubel , et al., “Catalytic janus Motors on Microfluidic Chip: Deterministic Motion for Targeted Cargo Delivery,” ACS Nano 6 (2012): 3383–3389. 22424213 10.1021/nn300413p

[14] C. Simó , M. Serra‐Casablancas , A. C. Hortelao , et al., “Urease‐Powered Nanobots for Radionuclide Bladder Cancer Therapy,” Nature Nanotechnology 19 (2024): 554–564, 10.1038/s41565-023-01577-y. PMC11026160 38225356

[15] D. Wang , B. Zhao , X. Li , et al., “Dexterous Electrical‐Driven Soft Robots With Reconfigurable Chiral‐Lattice Foot Design,” Nature Communications 14 (2023): 5067, 10.1038/s41467-023-40626-x. PMC10442442 37604806

[16] N. Ebrahimi , C. Bi , D. J. Cappelleri , et al., “Magnetic Actuation Methods in Bio/Soft Robotics,” Advanced Functional Materials 31 (2020): 2005137, 10.1002/adfm.202005137.

[17] Y. Cao , R. Xie , P. W. A. Schönhöfer , et al., “Permanent Magnetic Droplet–Derived Microrobots,” Science Advances 11 (2025): adw3172, 10.1126/sciadv.adw3172. PMC12239960 40632849

[18] X. Fan , Q. Chen , M. Li , et al., “Machining Swarf Formation–Inspired Fabrication of Ferrofluidic Helical Miniature Robots With Multimodal Locomotion Capability,” Science Advances 11 (2025): ads4411, 10.1126/sciadv.ads4411. PMC12227062 40614200

[19] X. Bao , F. Wang , J. Zhang , et al., “Real‐Time In Situ Magnetization Reprogramming for Soft Robotics,” Nature 645 (2025): 375–384, 10.1038/s41586-025-09459-0. 40759161 PMC12422961

[20] F. Heemeyer , Q. Boehler , M. Kim , et al., “Telesurgery and the Importance of Context,” Science Robotics 10 (2025): adq0192, 10.1126/scirobotics.adq0192. 40009655

[21] Y. Kim and X. Zhao , “Magnetic Soft Materials and Robots,” Chemical Reviews 122 (2022): 5317–5364, 10.1021/acs.chemrev.1c00481. 35104403 PMC9211764

[22] A. Cavallo , M. Madaghiele , U. Masullo , M. G. Lionetto , and A. Sannino , “Photo‐Crosslinked Poly(ethylene glycol) Diacrylate (PEGDA) Hydrogels From Low Molecular Weight Prepolymer: Swelling and Permeation Studies,” Journal of Applied Polymer Science 134 (2016): 44380, 10.1002/app.44380.

[23] L. Hertle , S. Sevim , J. Zhu , et al., “A Naturally Inspired Extrusion‐based Microfluidic Approach for Manufacturing Tailorable Magnetic Soft Continuum Microrobotic Devices,” Advanced Materials 36 (2024): 2402309, 10.1002/adma.202402309. 38780003

[24] L. Barth , M. Jung , R. Seemann , and K. Lienkamp , “3D printable Magnetic Soft Actuators–Ink Formulation, Rheological Characterization, and Hydrogel Actuator Prototypes,” Macromolecular Materials and Engineering 310 (2025): 2400431, 10.1002/mame.202400431.

[25] X. Zhao , F. R. Kolbinger , M. Distler , et al., “Portable Droplet‐Based Real‐Time Monitoring of Pancreatic Alpha‐Amylase in Postoperative Patients,” Biosensors and Bioelectronics 251 (2024): 116034, 10.1016/j.bios.2024.116034. 38359666

[26] X. Peng , Z. Janicijevic , S. Lemm , et al., “Shell Engineering in Soft Alginate‐Based Capsules for Culturing Liver Spheroids,” Biotechnology Journal 18 (2023): 2200365, 10.1002/biot.202200365. 36942860

[27] X. Peng , Z. Janicijevic , S. Lemm , et al., “Impact of Viscosity on Human Hepatoma Spheroids in Soft Core‐Shell Microcapsules,” Advanced Healthcare Materials 13 (2024): 2302609, 10.1002/adhm.202302609. 38227977 PMC11468952

[28] Y. Yan , C. Song , Z. Shen , et al., “Programming Structural and Magnetic Anisotropy for Tailored Interaction and Control of Soft Microrobots,” Communications Materials 3 (2024): 7, 10.1038/s44172-023-00145-5.

[29] P. Harder , N. Iyisan , Y. Wang , and B. Özkale , “A Soft Microrobot for Single‐Cell Transport, Spheroid Assembly, and Dual‐Mode Drug Screening,” Advanced Materials 38 (2025): 08807, 10.1002/adma.202508807. PMC13014025 41229318

[30] H. Xu , S. Wu , Y. Liu , et al., “3D Nanofabricated Soft Microrobots with Super‐Compliant Picoforce Springs as Onboard Sensors and Actuators,” Nature Nanotechnology 19 (2024): 494–503, 10.1038/s41565-023-01567-0. PMC11026159 38172430

[31] S. Gabler , J. Stampfl , T. Koch , et al., “Determination of the Viscoelastic Properties of Hydrogels Based on Polyethylene Glycol Diacrylate (PEG‐DA) and Human Articular Cartilage,” International Journal of Materials Engineering Innovation 1 (2009): 3–20.

[32] A. V. Samrot , C. S. Sahithya , and J. Selvarani A , “A Review on Synthesis, Characterization and Potential Biological Applications of Superparamagnetic Iron Oxide Nanoparticles,” Current Research in Green and Sustainable Chemistry 4 (2021): 100042, 10.1016/j.crgsc.2020.100042.

[33] D. T. Grob , N. Wise , O. Oduwole , and S. Sheard , “Magnetic Susceptibility Characterisation of Superparamagnetic Microspheres,” Journal of Magnetism and Magnetic Materials 452 (2018): 134–140, 10.1016/j.jmmm.2017.12.007.

[34] J. Gómez‐Pastora , I. H. Karampelas , E. Bringas , E. P. Furlani , and I. Ortiz , “Numerical Analysis of Bead Magnetophoresis From Flowing Blood in a Continuous‐Flow Microchannel: Implications to the Bead‐Fluid Interactions,” Scientific Reports 9 (2019): 7265, 10.1038/s41598-019-43827-x. 31086252 PMC6514169

[35] J. Ruan , W. Zhang , C. Zhang , N. Li , J. Jiang , and H. Su , “A Magnetophoretic Microdevice for Multi‐Magnetic Particles Separation Based on Size: A Numerical Simulation Study,” Engineering Applications of Computational Fluid Mechanics 16 (2022): 1781–1795, 10.1080/19942060.2022.2109064.

[36] F. Ongaro , S. Pane , S. Scheggi , and S. Misra , “Design of an Electromagnetic Setup for Independent Three‐dimensional Control of Pairs of Identical and Nonidentical Microrobots,” IEEE Transactions on Robotics 35 (2019): 174–183, 10.1109/tro.2018.2875393.

[37] J. D. Keuning , J. de Vriesy , L. Abelmanny , and S. Misra , “Image‐Based Magnetic Control of Paramagnetic Microparticles in Water,” In 2011 IEEE/RSJ International Conference on Intelligent Robots and Systems (IEEE, 2011): 421–426.

[38] M. B. Browning , S. N. Cereceres , P. T. Luong , et al., “Determination of the in Vivo Degradation Mechanism of PEGDA Hydrogels,” Journal of Biomedical Materials Research Part A 102 (2014): 4244–4251, 10.1002/jbm.a.35096. 24464985 PMC4112173

[39] Z. Stillman , B. M. Jarai , N. Raman , P. Patel , and C. A. Fromen , “Degradation Profiles of Poly(ethylene glycol) Diacrylate (PEGDA)‐Based Hydrogel Nanoparticles,” Polym Chem 11 (2020): 568–580, 10.1039/c9py01206k. 33224282 PMC7678750

[40] X. Zhao , R. Illing , P. Ruelens , et al., “Coexistence of Fluorescent Escherichia coli Strains in Millifluidic Droplet Reactors,” Lab on a Chip 21 (2021): 1492–1502, 10.1039/d0lc01204a. 33881032

[41] K. Li , Y. He , X. Jin , K. Jin , and J. Qian , “Reproducible Extracellular Matrices for Tumor Organoid Culture: Challenges and Opportunities,” Journal of Translational Medicine 23 (2025): 497, 10.1186/s12967-025-06349-x. 40312683 PMC12044958

[42] S. Gervasoni , N. Pedrini , T. Rifai , et al., “A human‐scale Clinically Ready Electromagnetic Navigation System for Magnetically Responsive Biomaterials and Medical Devices,” Advanced Materials 36 (2024): 2310701, 10.1002/adma.202310701. 38733269

[43] F. C. Landers , L. Hertle , V. Pustovalov , et al., “Clinically Ready Magnetic Microrobots for Targeted Therapies,” Science 390 (2025): 710–715. 41231973 10.1126/science.adx1708

[44] X. Peng , Z. Janicijevic , L. R. Loureiro , et al., “Microphysiological Solid Tumor Models in Hydrogel Beads for CAR T Cell Immunotherapy Evaluation,” Advanced Science 12 (2025): 08267, 10.1002/advs.202508267. PMC12591166 40704837

[45] G. Bradski , “The OpenCV Library,” Dr Dobb's Journal: Software Tools for the Professional Programmer 25 (2000): 120–123.

[46] A. Doctor , M. Laube , S. Meister , et al., “Combined PET Radiotracer Approach Reveals Insights into Stromal Cell‐Induced Metabolic Changes in Pancreatic Cancer In Vitro and In Vivo,” Cancers (Basel) 16 (2024): 3393, 10.3390/cancers16193393. 39410013 PMC11475921

