# Supplementary material for: Soft, Degradable, and Magnetic Microcarriers for Encapsulation and Guided Transport of Drugs and 3D Spheroids
Source: Adv Mater. 2026 Jun 17;38(41):e73735. doi: 10.1002/adma.73735 (PMC13393984; doi:10.1002/adma.73735)
Supplement: Supplementary file 1 — Supporting File 1: adma73735‐sup‐0001‐SuppMat.docx. [file ADMA-38-e73735-s002.docx]

**Supporting Information**

Supporting Information is available from the Wiley Online Library or from the author.

Supporting Information

**Soft, Degradable, and Magnetic Microcarriers for Encapsulation and Guided Transport of Drugs and 3D Spheroids**

Xuan Peng, Lulu Song, Daryna Mruga, Veronika Bakhmat, Sergei Dzyadevych, Lin Guo, Shahrukh Shakeel, Rico Illing, Olha Bezsmertna, Xiaotao Wang, Nicholas R. Posselli1, Sarthak Misra, Sandra Hauser, Jens Pietzsch, Klaus Kopka, Denys Makarov, Željko Janićijević, Xinne Zhao, Larysa Baraban*

**Note 1.**

The magnetic moment of an ensemble of non-interacting superparamagnetic particles, $\vec{m}_{B},$is determined by the product of the number of particles, *N*, volume of the individual particle, $V_{SP}$, strength of the applied magnetic field, $\vec{B}/\mu_{0}$, and magnetic susceptibility of the beads, $\chi_{SP}$:

|  | $\vec{m}_{B}=N V_{SP}\frac{\chi_{SP}}{\mu_{0}}\vec{B}$ |  |
| --- | --- | --- |

μ_0_ = 4π × 10^−7^ (T m A^−1^) is the permeability of vacuum.

For a sufficiently strong dipole-dipole interaction between superparamagnetic particles, the ensemble could develop a remanent magnetic moment, $\vec{m}_{R}$. Hence, the total moment of the ensemble is $\vec{m}_{0}=\vec{m}_{R}+\vec{m}_{B}$. When the carried is exposed to an inhomogeneous magnetic field, there will be a driving magnetic force, which will bring this carried into motion:

$$\vec{F}_{mag}=\nabla\left( \vec{m}_{0}\vec{B} \right)$$

For the case, when the distribution of superparamagnetic particles within the carrier is inhomogeneous (e.g., presence of clustering or chaining), the total moment will be coordinate-dependent. For a homogeneous distribution of particles within the carrier, $\vec{F}_{mag}=\vec{m}_{0}\nabla\vec{B}=\left( \vec{m}_{R}+\zeta\chi_{SP}\vec{B} \right)\nabla\vec{B}$. In our setup, the field gradient is proportional to the strength of the applied magnetic field. Hence, $F_{mag}\sim m_{R}B+\zeta\chi_{SP}B^{2}$, with *B* being the strength of the magnetic field.

We would like to note the following: At lower particle concentrations where the interparticle interaction is negligible, the magnetic response is mainly governed by the field-induced magnetization of superparamagneetic dynaparticles, $\vec{m}_{B}$, leading to the expected quadratic nonlinear dependence of the velocity on the magnetic field. At higher concentration of magnetic particles per carrier, the approximation of non-interacting particles can become violated. In this case, within the mean-field model, the magnetic moment of the ensemble, exposed to an external magnetic field, will scale with the concentration nonlinearly with a typical exponent between 1 and 2. This is a direct consequence of the development of a finite magnetization, $\vec{m}_{R}$, even at remanence when the strength of the dipole-dipole interaction becomes comparable with or exceeds the thermal activation energy. In this case, the expression for the magnetic force will acquire an additional term reflecting that this finite magnetization can interact with the gradient magnetic field. This will add a velocity term, which is linear with the applied magnetic field for our experimental setup where the field gradient is proportional to the magnetic field.

The effect of an inhomogeneous particle distribution within the carrier can enable this linear contribution. Indeed, for the case of an inhomogeneous particle distribution, there could appear regions where the local particle concentration could become sufficiently high to develop a finite remanent magnetic moment even if the averaged particle concentration is low. In such a case, the velocity of the carrier will contain two terms: one is quadratic in the field strength (originates from the regions, where the dynaparticle concentration is low) and another one is linear in the field strength (originates from the regions, where the dynaparticle concentration is high).

We note that for an ensemble of dynaparticles with a typical magnetization of 10^4^ A/m and diameter of 1 μm, the critical concentration where the collective behavior is observed corresponds to less than 1% v/v. In this respect, the field-driven dynamics of the microcarrier strongly depends on the actual dependence of the field-dependence of the magnetic moment. In our experiments, this dependence is further affected by an inhomogeneity in the distribution of dynaparticles within the carrier.

For instance, if the concentration and local distribution of dynaparticles within the carrier is such that the remanent magnetization will dominate the magnetic moment of the carrier at low fields, the velocity will scale linearly with the field. As our experiments are carried out at rather high concentrations of dynaparticles and their clustering cannot be avoided, the final velocity dependence on the magnetic field is very sensitive to the heterogeneity of the composite.

Additionally, asymmetric particle distributions within the hydrogel matrix may contribute to the observed behavior. In particular, local particle heterogeneity or preferential particle accumulation could generate anisotropic magnetic responses, potentially affecting both the magnitude and directionality of carrier motion. Similar effects of magnetic anisotropy and particle organization influencing collective magnetic behavior have recently been reported in soft microrobotic systems.


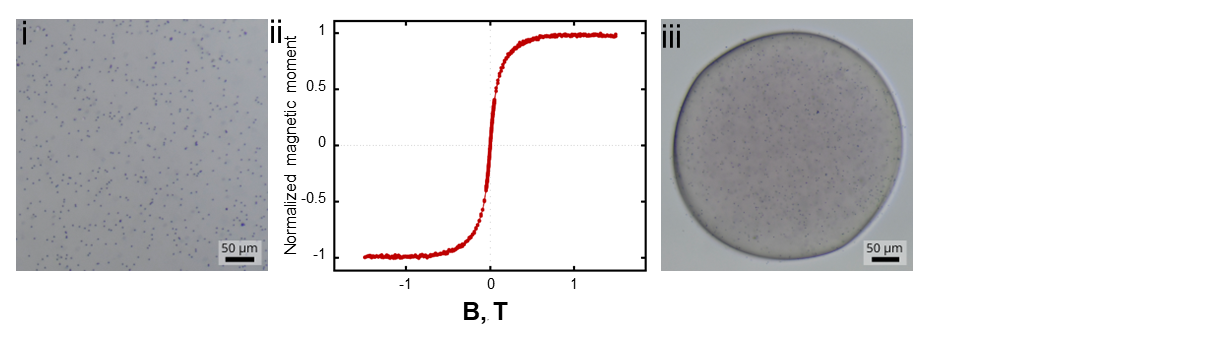


**Figure S1.** (i) Optical image of superparamagnetic Dynaparticles (diameter of 1 μm) in solution. (ii) Magnetic hysteresis loop of a dried Dynaparticles solution measured by vibrating sample magnetometry (VSM) at room temperature. (iii) Optical image of Dynaparticles encapsulated in a polymeric bead at concentration of 0.05%, UV mean intensity = 34 mW cm^-2^, irradiation time 1.22 s.


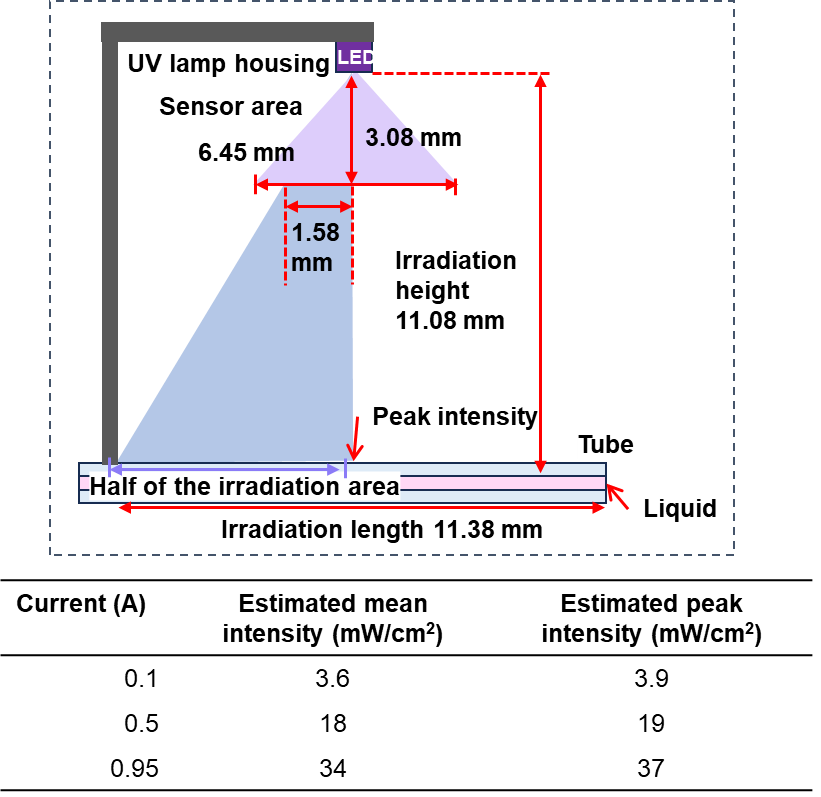


**Figure S2.** Illustration of the UV power measurement and UV intensities estimated on the surface of a tube using a custom MATLAB script.


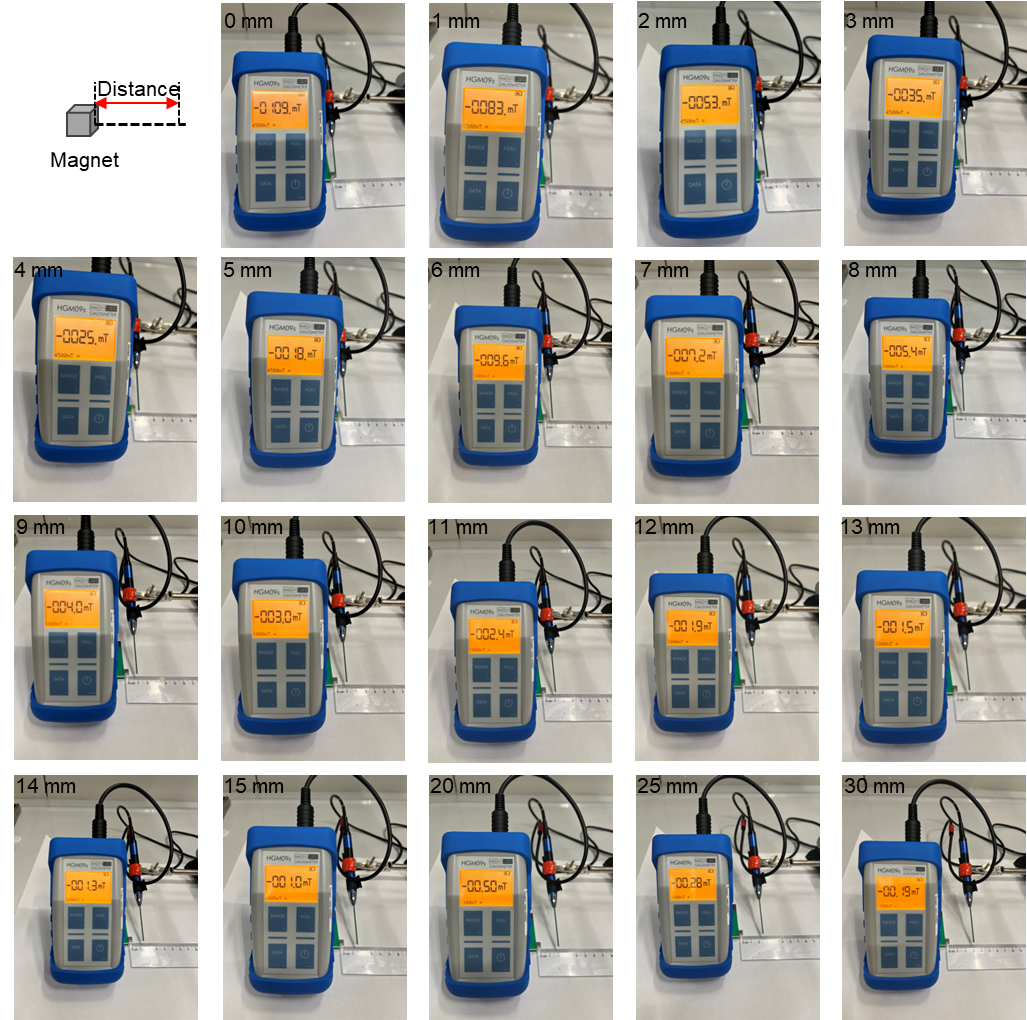


**Figure S3.** Measurement procedure for determining the magnetic field decay of a cube magnet. Schematic showing how the magnetic field of the cube magnet was measured as a function of distance using a handheld Gaussmeter.


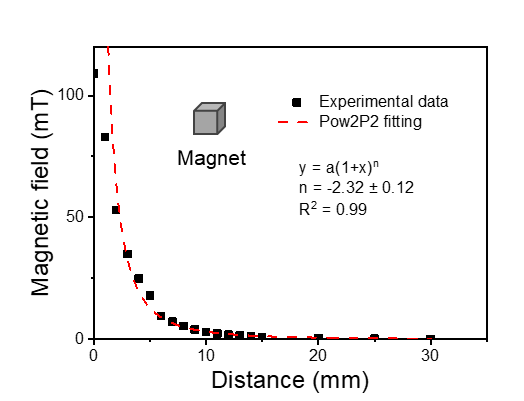


**Figure S4.** Magnetic field decay data and the fitted two-parameter power-law model, based on the measurement procedure illustrated in Figure S3.


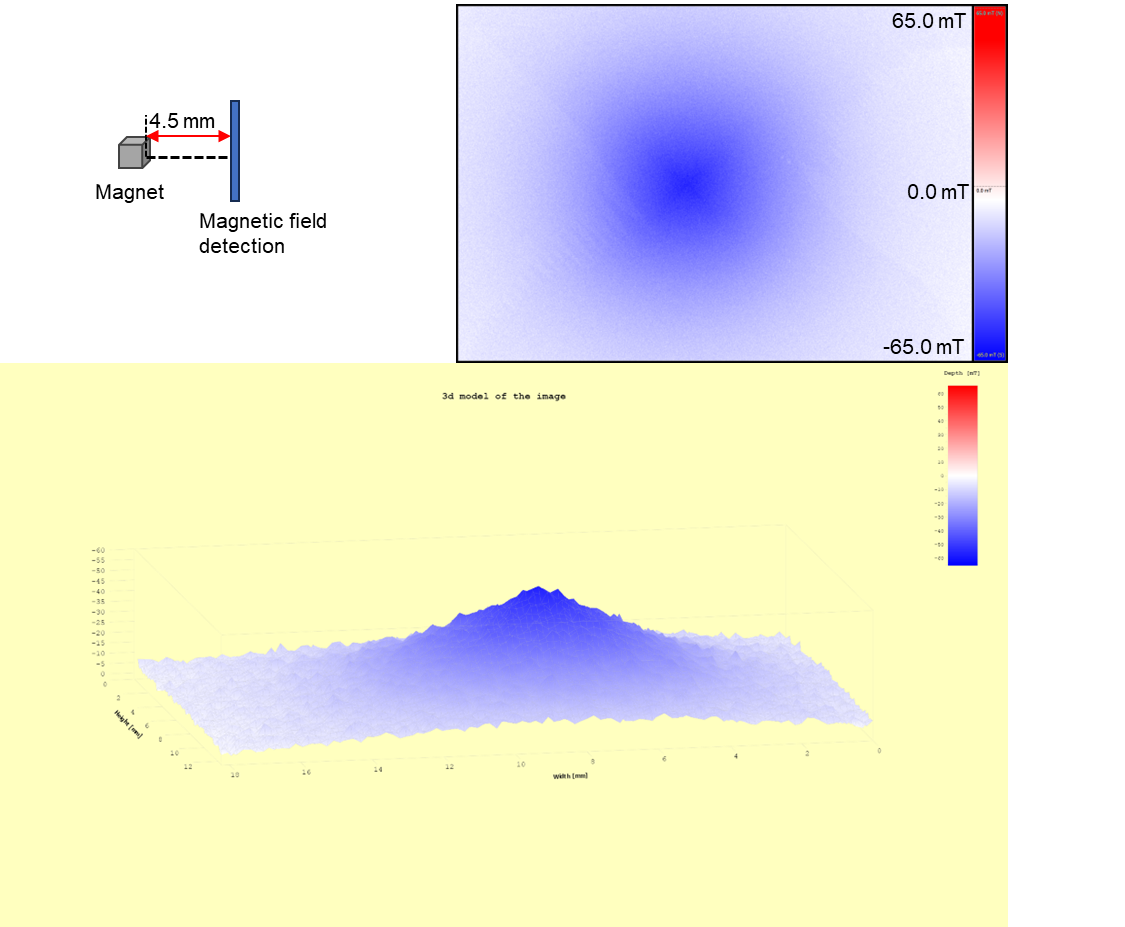


**Figure S5.** Magnetic field distribution at the cubic magnet surface measured using CMOS-MagView. Top left: Illustration of the setup used to measure the magnetic field distribution at the small surface of the cubic magnet. Top right: Two-dimensional magnetic field intensity map measured using CMOS-MagView, which provides a surface field distribution over an approximately 4.5 mm radius distance from the magnet’s surface. Bottom: Three-dimensional rendering of the measured surface field distribution.


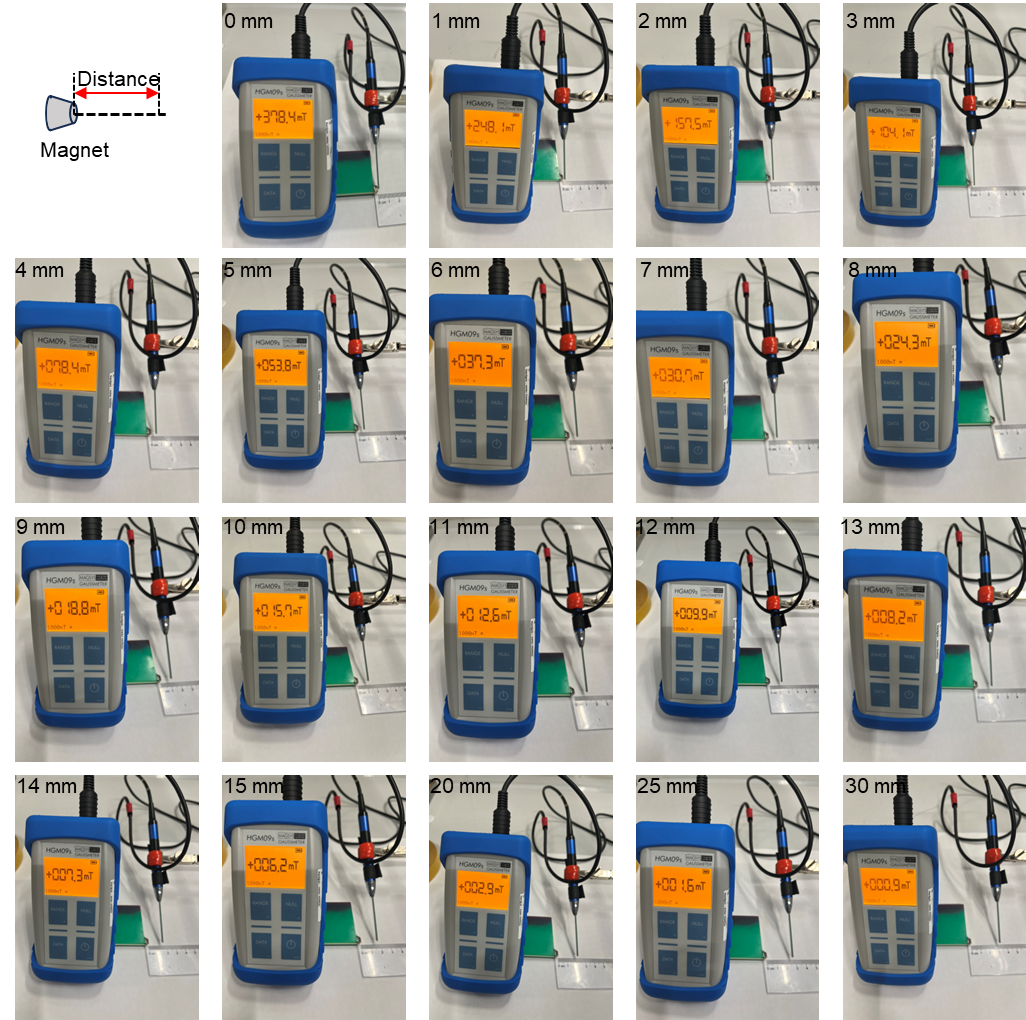


**Figure S6.** Measurement procedure for determining the magnetic field decay of a conical magnet. Schematic showing how the magnetic field of the cube magnet was measured as a function of distance using a handheld Gaussmeter.


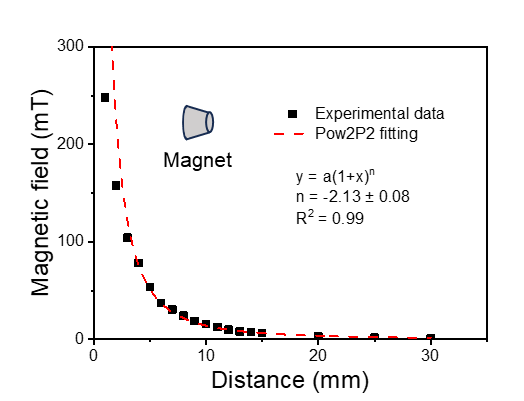


**Figure S7.** Magnetic field decay data and the fitted two-parameter power-law model, based on the measurement procedure illustrated in Figure S6.


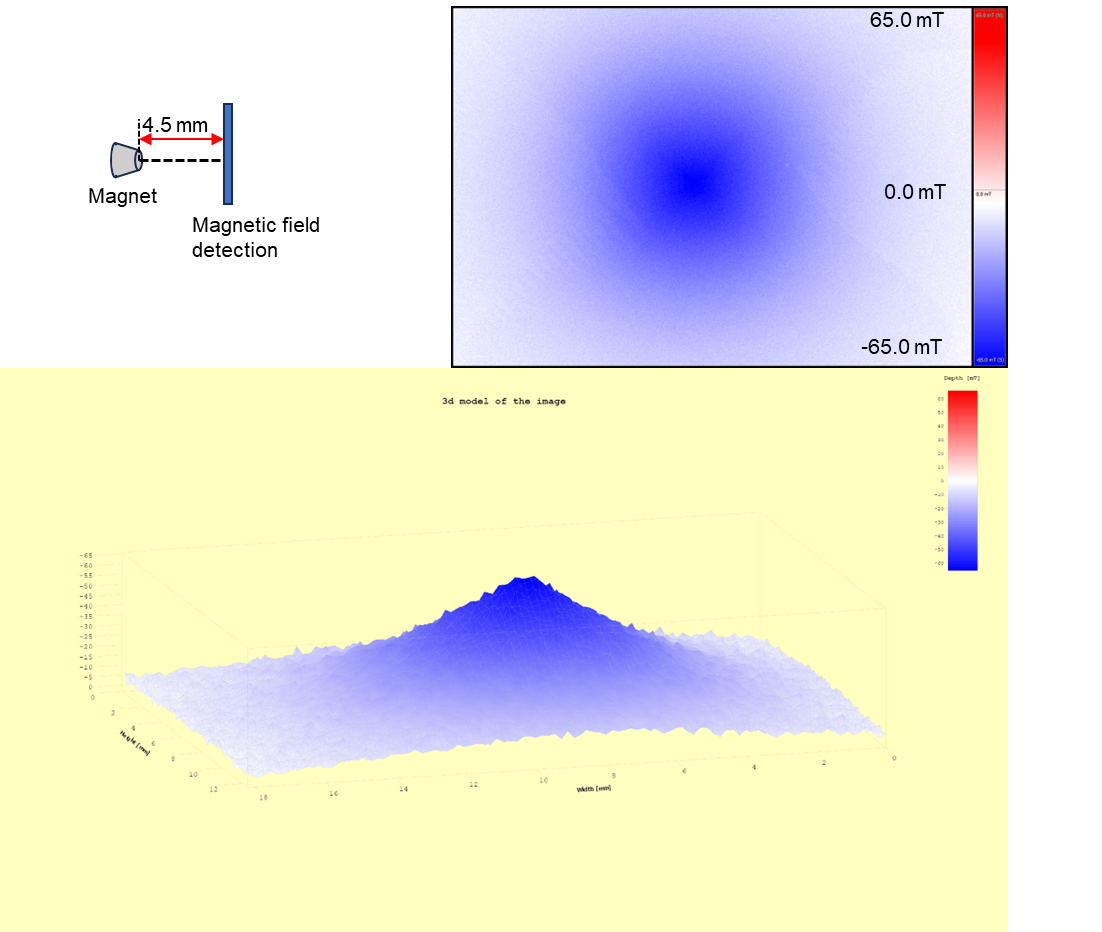


**Figure S8.**  Magnetic field distribution at the small surface of the conical magnet measured using CMOS-MagView. Top left: Illustration of the setup used to measure the magnetic field distribution at the small surface of the conical magnet. Top right: Two-dimensional magnetic field intensity map measured using CMOS-MagView, which provides a surface field distribution over an approximately 4.5 mm region distance from the magnet’s surface. Bottom: Three-dimensional rendering of the measured surface field distribution.


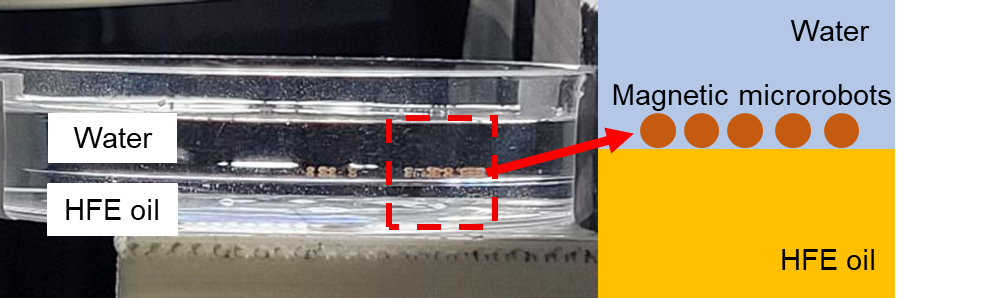


**Figure S9.** Illustration of the setup used to study the velocity of microrobots. Magnetic microrobots were loaded in a petri dish containing water (top) and HFE oil (bottom).


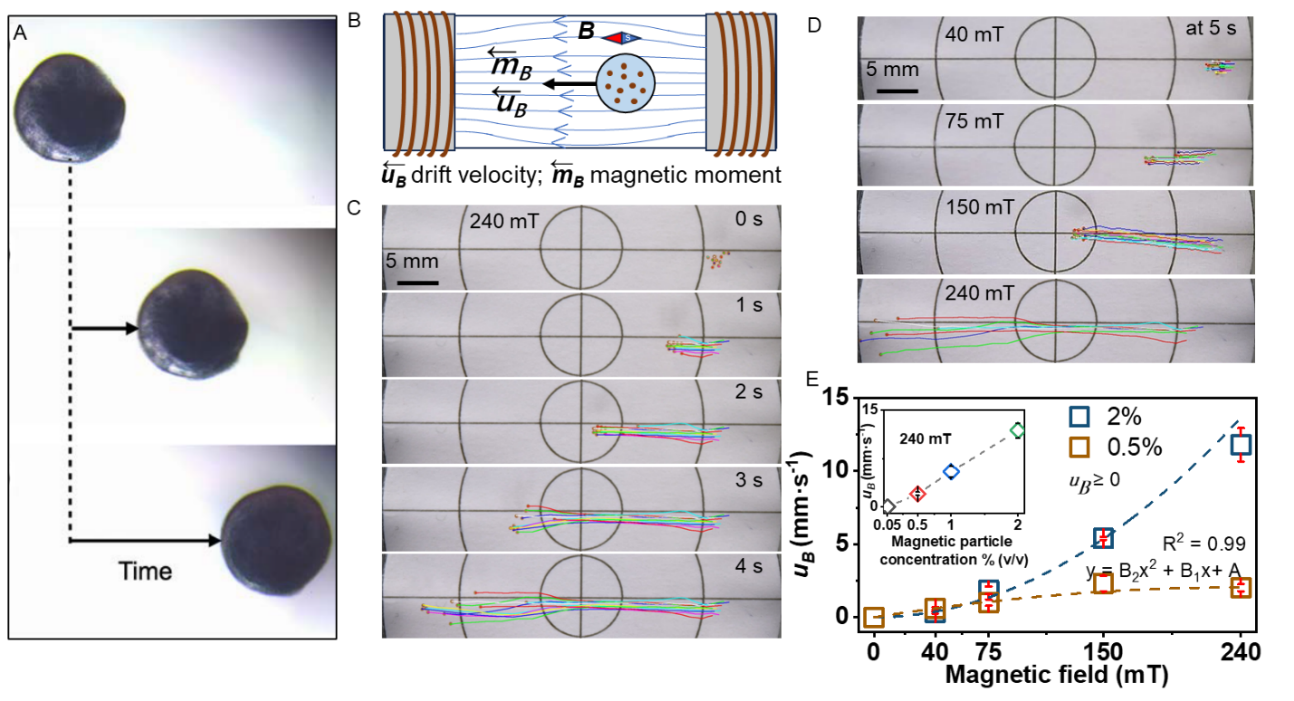


**Figure S10.** Motion of beads in a gradient magnetic field. (A) Snapshots of a movie revealing a controlled displacement of a magnetic bead in a gradient field of a permanent magnet. The magnet is located at the right outside the field of view of the microscope. (B) Schematic of the velocity measurement experiment when magnetic beads are exposed to a gradient magnetic field. (C) Beads tracking over time under the applied magnetic field of 240 mT (gradient: 2 T m^-1^). (D) Tracked paths of the same beads at the 5-s time stamp under different magnetic field strengths (40, 75, 150, and 240 mT) and corresponding gradients (0.3, 0.6, 1.3, and 2 T m^-1^), respectively. (E) Average velocities obtained from the tracking of different magnetic microrobots containing 0.5–2% (v/v) of magnetic particles under different magnetic field strengths. Inset: Average velocities of magnetic microrobots containing 0.5–2% (v/v) of magnetic particles measured under a magnetic field strength of 240 mT. The diameter of the bead is 500 μm. The microrobots contain 2% (v/v) of magnetic particles in panels A, C, and D. UV mean intensity = 34 mW cm^-2^, UV exposure time: 1.22 s.


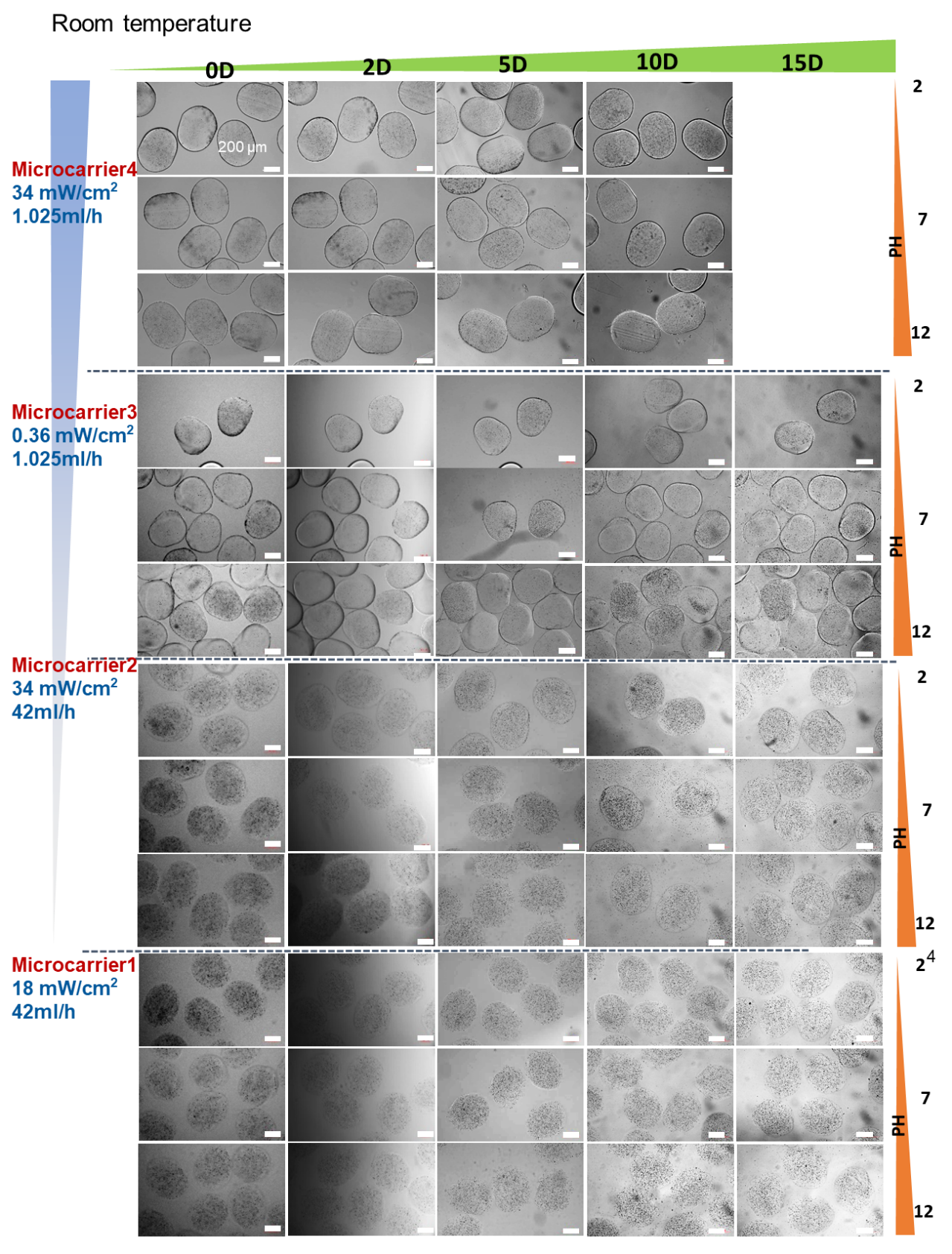


**Figure S11.** Morphology of magnetic PEGDA700 microcarriers incubated at room temperature under acidic (pH 2), neutral (pH 7), and alkaline (pH 12) conditions. The microcarriers remained stable, with UV mean intensity and irradiation time having minimal impact on stability. Scale bar = 200 μm. The microcarrier fabrication conditions are described in Table S1.


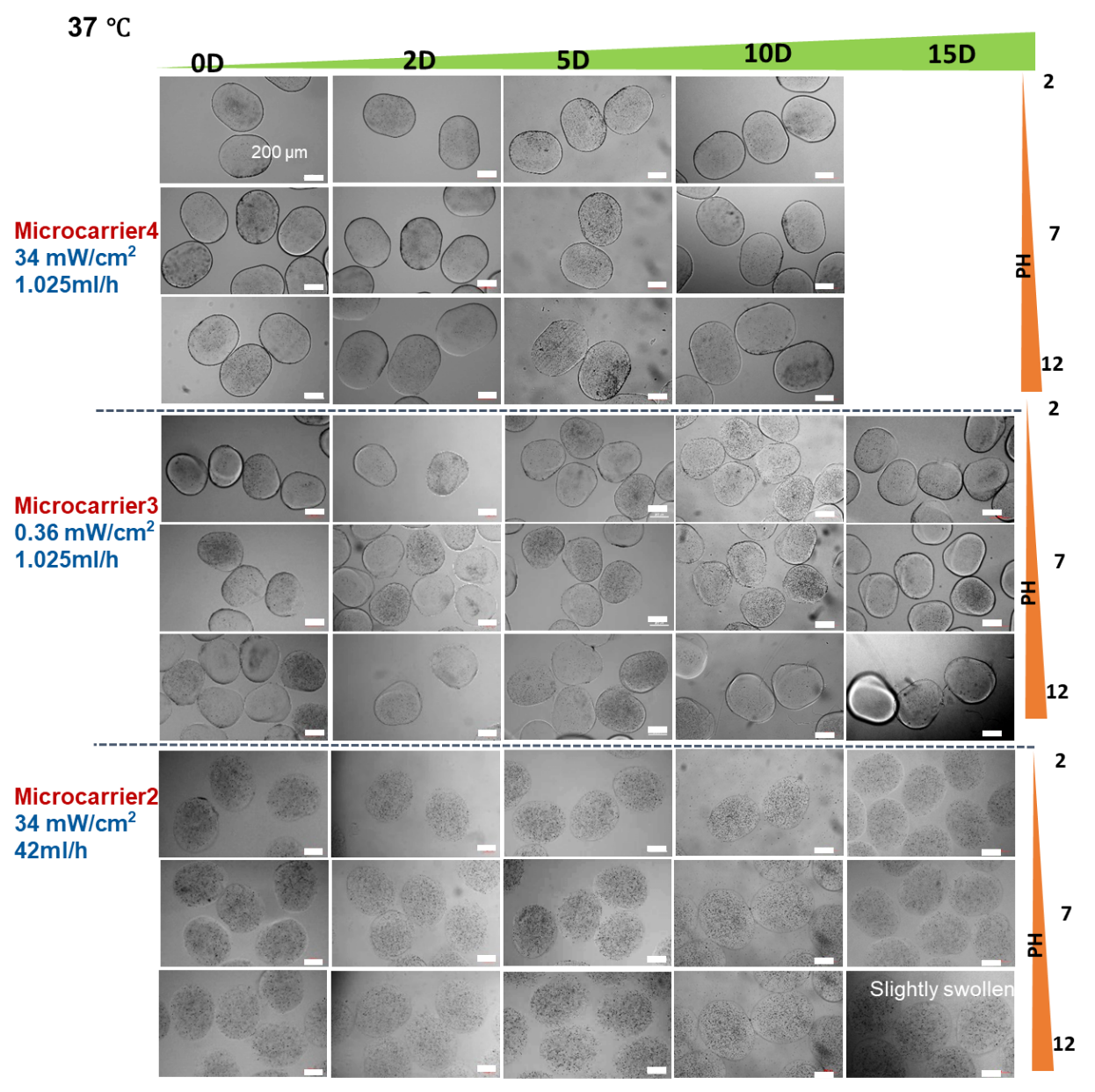


**Figure S12.** Morphology of magnetic PEGDA700 microcarriers incubated at 37 ℃ under acidic (pH 2), neutral (pH 7), and alkaline (pH 12) conditions. Microcarriers generated under UV mean intensity (~30 mW cm^-2^) with a short irradiation time (~0.2 s) appeared slightly swollen under alkaline conditions, but remained more stable under neutral and acidic conditions. Scale bar = 200 μm. The microcarrier fabrication conditions are described in Table S1.

**
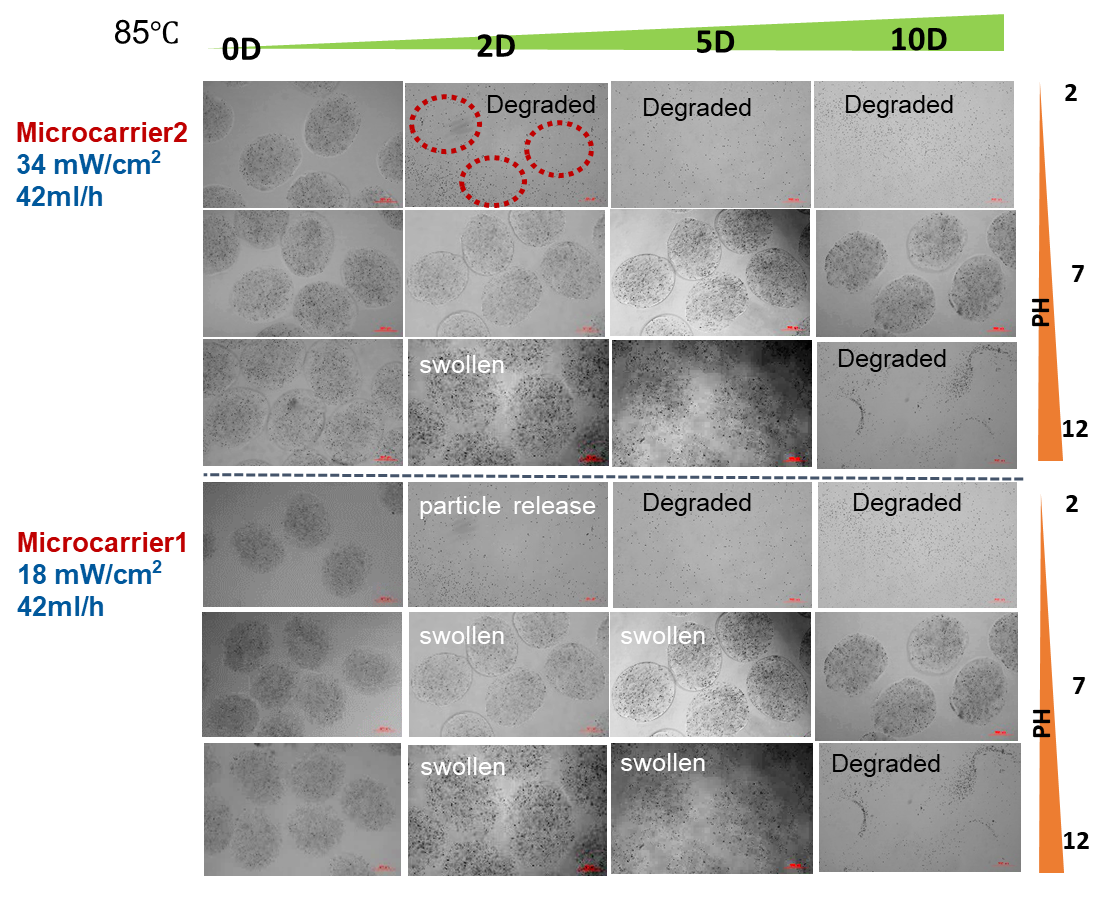
**

**Figure S13.** Morphology of magnetic PEGDA700 microcarriers incubated at 85 °C under acidic (pH 2), neutral (pH 7), and alkaline (pH 12) conditions. The microcarriers degraded much faster under acidic and alkaline conditions, while exhibiting markedly better stability in neutral conditions. The microcarrier fabrication conditions are described in Table S1.


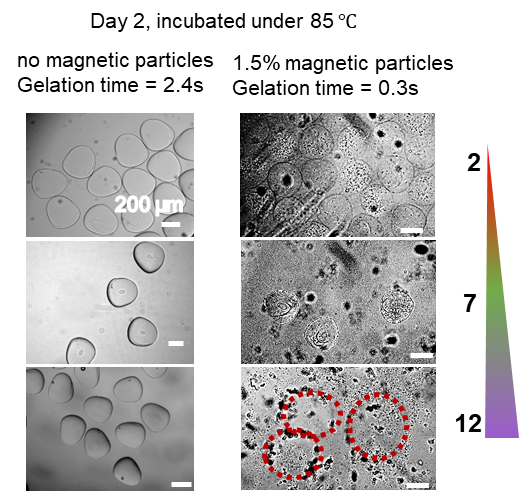


Figure S14. Influence of the gelation time and concentration of magnetic particles on the degradation of soft magnetic microrobots. Samples were prepared under mean UV mean intensity of 5.4 mW cm^-2^.


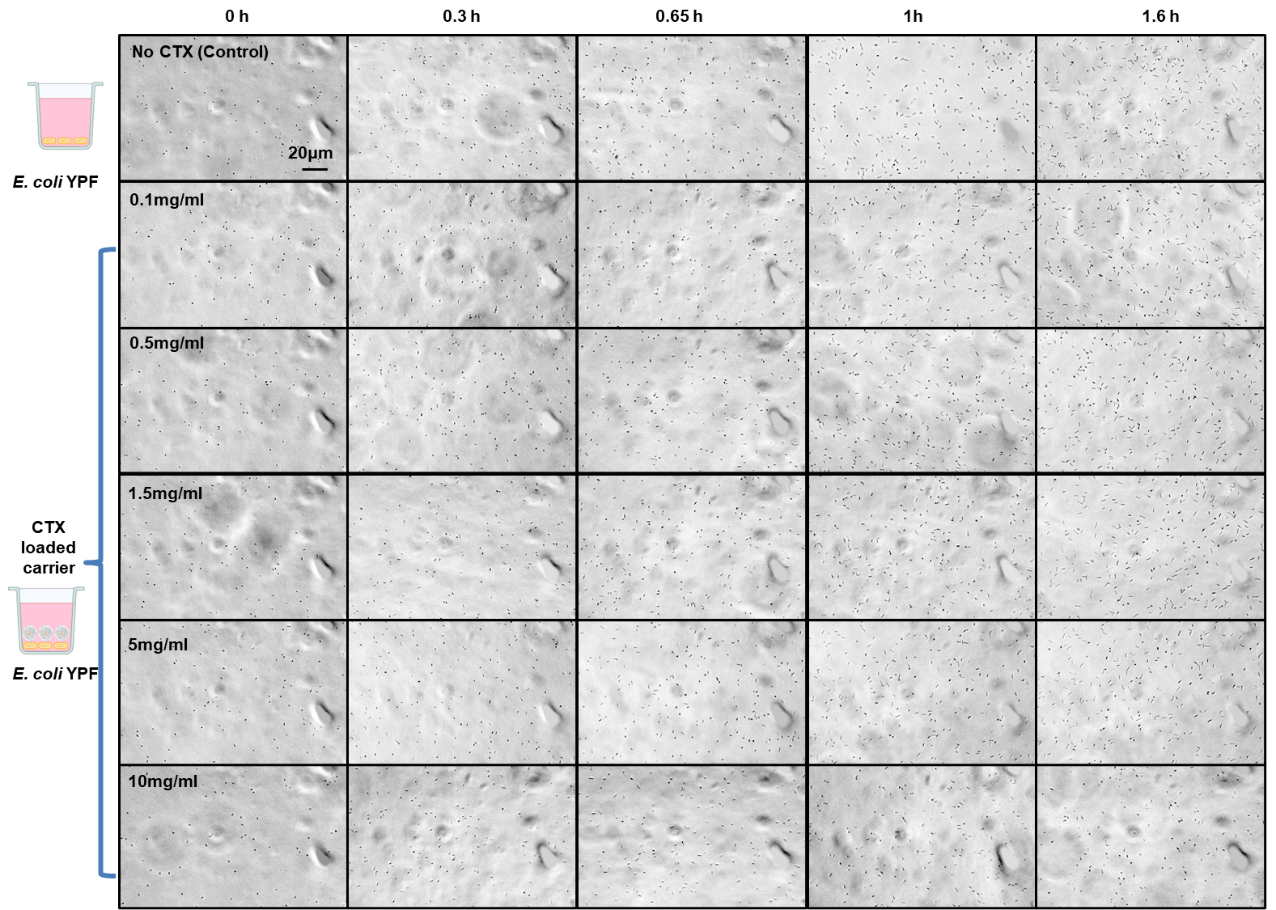


Figure S15. Proliferation and growth of *E. coli* YFP in the presence of magnetic carriers with different concentrations of CTX (0.1, 0.5, 1.5, 5, and 10 mg mL^-1^) during 1-1.6 h of culturing. No significant difference were observed.


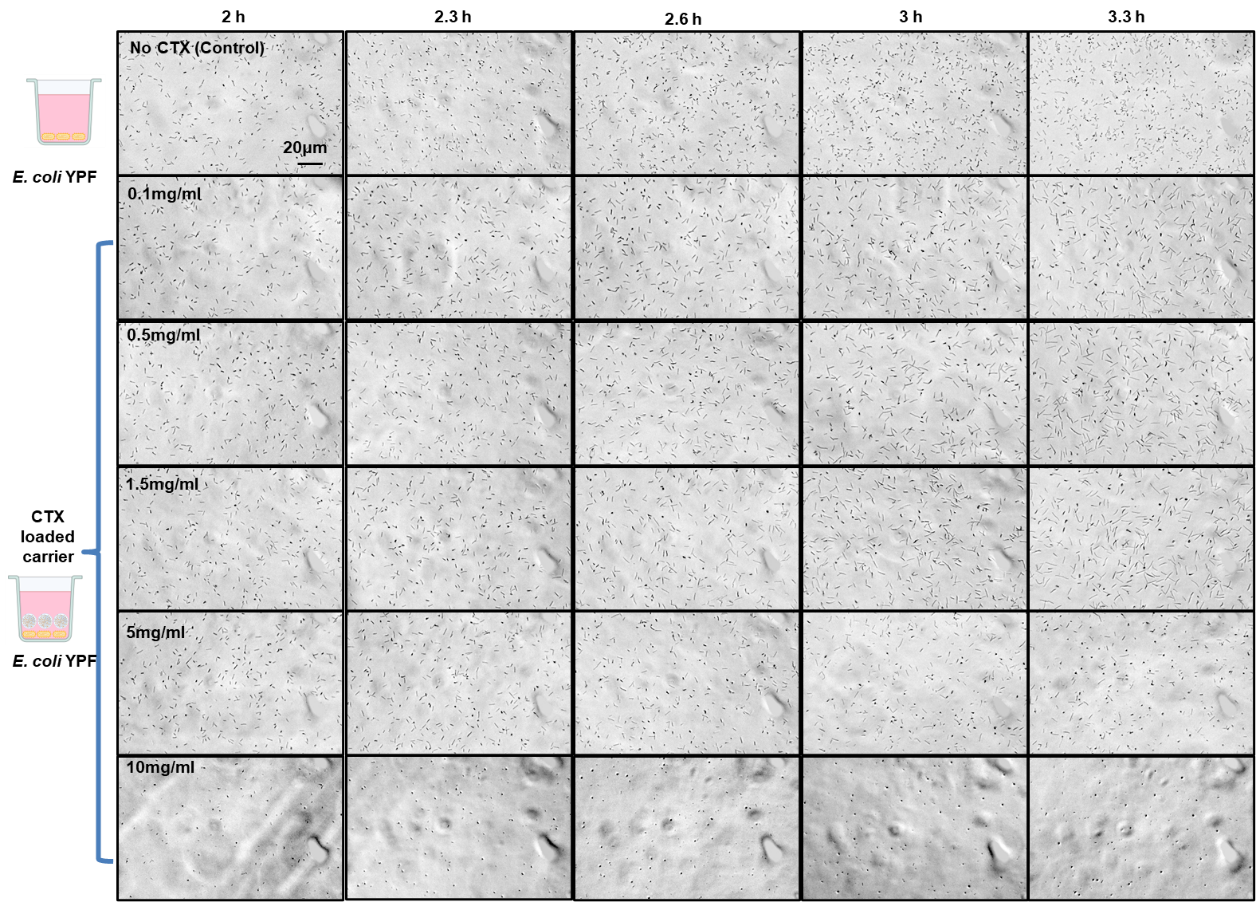


Figure S16. Proliferation and growth of *E. coli* YFP in the presence of magnetic carriers with different concentrations of CTX (0.1, 0.5, 1.5, 5, and 10 mg mL^-1^) during 2-3.3 h of culturing. *E. coli* were killed under the microcarrier loaded with a high concentration of CTX.


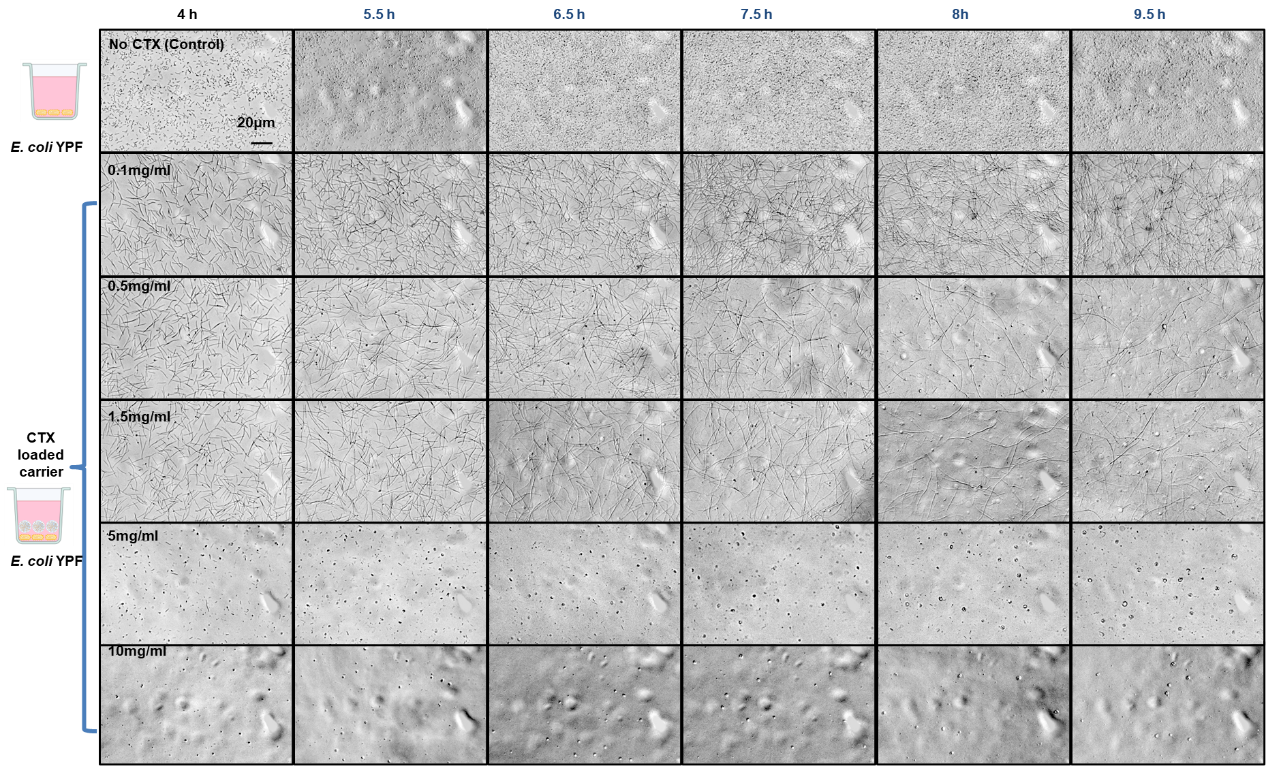


Figure S17. Proliferation and growth of *E. coli* YFP in the presence of magnetic carriers with different concentrations of CTX (0.1, 0.5, 1.5, 5, and 10 mg mL^-1^) during 4-9.5 h of culturing. Under the conditions of 0.1 μg mL^-1^ to 1.5 μg mL^-1^, cells exhibited a tendency to form filaments.


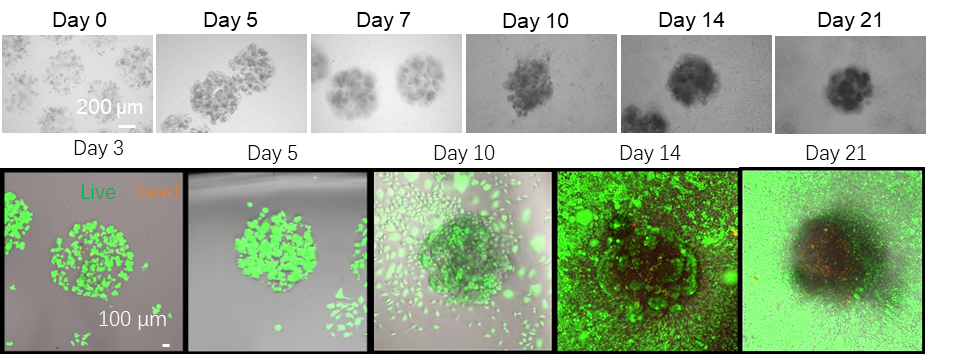


Figure S18. L929 cell proliferation and Live/Dead staining in PEGDA microcarriers without magnetic particles.


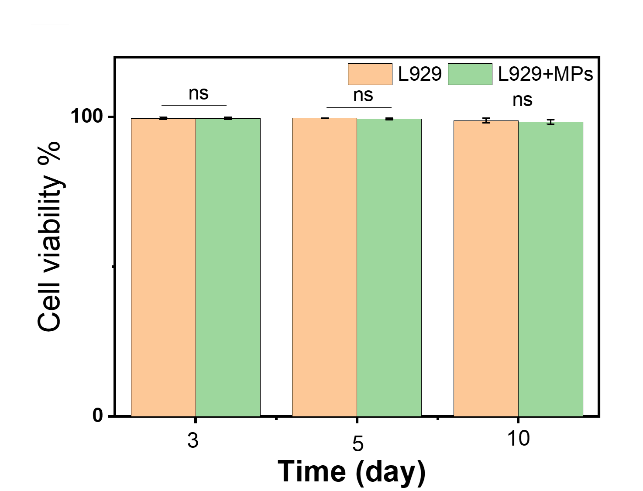


Figure S19. Cell viability in PEGDA Microcarriers with (L929+MPs) and without (L929) magnetic particles


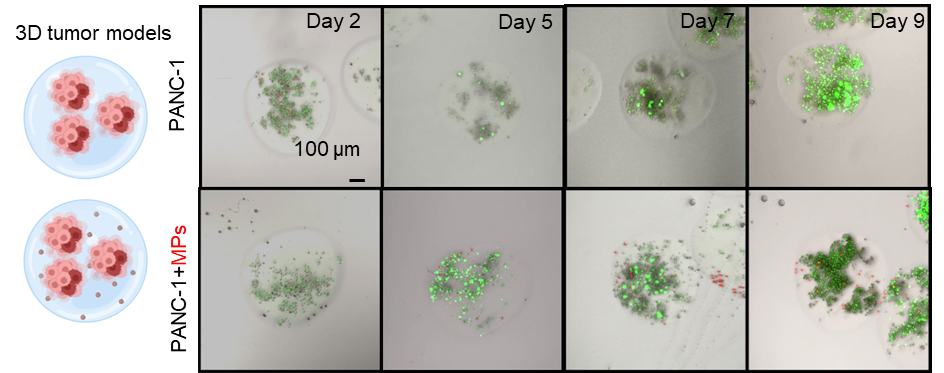


Figure S20. *In vitro* 3D PANC-1 tumor model in PEGDA microcarriers generated with & without magnetic particles and corresponding Live/Dead staining.

**Table S1.** Different PEGDA hydrogel microcarrier generation parameters.

| Materials | No. | Estimated mean intensity (mW cm^-2^) | Flow rate for droplet generation (ml h^-1^) | Flow rate for hydrogel generation (ml h^-1^) |
| --- | --- | --- | --- | --- |
| 10 % (w/v) PEGDA Mn=700;  0.1 % (w/v) LAP; 1 % (V/V) magnetic particles (1 μm)  HFE oil | 1 | 18 | 40 (oil) : 2 (water) | 42 |
|  | 2 | 34 |  | 42 |
|  | 3 | 0.36 |  | 1.025 |
|  | 4 | 34 |  | 1.025 |
| 10 % (w/v) PEGDA Mn=6000;  0.1 % (w/v) LAP; 2 % (v/v) magnetic particles (10 μm)  Mineral oil |  | 230 | 6.66 (oil) : 0.333 (water) | 6.993 |
| 7 % (w/v) PEGDA Mn=6000;  0.1% (w/v) LAP; 2% (v/v) magnetic particles (10 μm)  Mineral oil |  | 230 | 6.66 (oil) : 0.333 (water) | 6.993 |

**References**

[1] Y. Cao, R. Xie, P. W. A. Schönhöfer, R. Burdis, R. Wang, R. Sun, K. Xie, J. Zou, X. Song, Q. Y. Lau, J. Lin, J. A. Kim, D. Georgiev, J. Tang, H.-C. Ng, O. Bibikova, Y. Zuo, X. L. Lu, S. C. Glotze and M. M. Stevens, Permanent magnetic droplet–derived microrobots, Sci. Adv. 2025, 11, eadw3172. https://doi.org/10.1126/sciadv.adw3172.

[2] Y. Yan, C. Song, Z. Shen, Y. Zhu, X. Ni, B. Wang, M. G. Christiansen, S. Stavrakis, J.. S. Lintuvuori, B. Chen, A. deMello, S. Schuerleet al. Programming structural and magnetic anisotropy for tailored interaction and control of soft microrobots. Commun. Eng. 2024, 3, 7. https://doi.org/10.1038/s44172-023-00145-5.
